# Regulation of ribonucleotide synthesis by the *Pseudomonas aeruginosa* two-component system AlgR in response to oxidative stress

**DOI:** 10.1038/s41598-017-17917-7

**Published:** 2017-12-20

**Authors:** Anna Crespo, Lucas Pedraz, Marc Van Der Hofstadt, Gabriel Gomila, Eduard Torrents

**Affiliations:** 1 Bacterial Infections: Antimicrobial Therapies, Institute for Bioengineering of Catalonia (IBEC), The Barcelona Institute of Science and Technology, Barcelona, Spain; 2 Nanoscale Bioelectrical Characterization, Institute for Bioengineering of Catalonia (IBEC) and University of Barcelona, The Barcelona Institute of Science and Technology, Barcelona, Spain

## Abstract

Ribonucleotide reductases (RNR) catalyze the last step of deoxyribonucleotide synthesis, and are therefore essential to DNA-based life. Three forms of RNR exist: classes I, II, and III. While eukaryotic cells use only class Ia RNR, bacteria can harbor any combination of classes, granting them adaptability. The opportunistic pathogen *Pseudomonas aeruginosa* surprisingly encodes all three classes, allowing it to thrive in different environments. Here we study an aspect of the complex RNR regulation whose molecular mechanism has never been elucidated, the well-described induction through oxidative stress, and link it to the AlgZR two-component system, the primary regulator of the mucoid phenotype. Through bioinformatics, we identify AlgR binding locations in RNR promoters, which we characterize functionally through EMSA and physically through AFM imaging. Gene reporter assays in different growth models are used to study the AlgZR-mediated control on the RNR network under various environmental conditions and physiological states. Thereby, we show that the two-component system AlgZR, which is crucial for bacterial conversion to the mucoid phenotype associated with chronic disease, controls the RNR network and directs how the DNA synthesis pathway is modulated in mucoid and non-mucoid biofilms, allowing it to respond to oxidative stress.

## Introduction

*Pseudomonas aeruginosa* is a ubiquitous environmental Gram-negative bacterium, but it can also be a dangerous and adaptable opportunistic pathogen. In particular, it is known to cause severe chronic lung infections in immunocompromised patients and other at-risk groups. In cystic fibrosis (CF) patients, this infection is associated with a poor prognosis, leading to severely impaired lung function and an increased risk of respiratory failure, and is the primary cause of morbidity and mortality^1^. *P. aeruginosa* initially colonizes the CF lung in a non-mucoid form (characterized by non-detectable alginate production and causing an asymptomatic infection). However, at later stages of lung colonization, *P. aeruginosa* switches its phenotype to a mucoid alginate-overproducer variant, leading to rapid pulmonary deterioration^2,3^.

Alginate production protects *P. aeruginosa* from phagocytosis, antibiotic penetration, and desiccation^4,5^, but it is also an energy-intensive process and is therefore closely regulated and activated only when a chronic infection reaches a critical point. It involves a large number of enzymes and precursor substrates. Of particular relevance is the *algD* (and consecutive genes) operon, encoding the main enzymes for alginate production, and the *algC* gene from the *algC-argB* operon, a multifunctional enzyme required for several pathways including alginate biosynthesis and LPS production^6^. These genes are controlled by products of the *algU/mucABCD* operon; their transcription is directed by the alternative sigma factor AlgU (sigma E), which is commonly sequestered by the anti-sigma factor MucA. It has been reported that several types of cellular stress can induce proteolytic degradation of MucA, releasing AlgU and transiently activating alginate synthesis^7^, but the stable mucoid phenotype is generated through the selection of mutations in the regulatory genes, usually in *mucA* ^8,9^.

Apart from the *algD* and *algC* operons, the AlgU sigma factor regulates the transcription of the *fimS*(*algZ*)-*algR* operon, which encodes the AlgZR two-component system^10,11^. In this system, FimS is the membrane kinase that can detect an unknown environmental signal and accordingly modulate the phosphorylation of AlgR. In turn, AlgR is the transcriptional factor that, depending on its phosphorylation state, regulates all aspects of alginate biosynthesis (controlling the *algD* and *algC* operons), as well as several aspects of anaerobic metabolism, type IV pili formation, rhamnolipid biosynthesis, type III secretion, and cyanide and nucleotide synthesis^12,13^. Furthermore, it has recently been reported to bind with high affinity to 157 loci in the *P. aeruginosa* genome^12^. Many of the functions regulated by the AlgZR system are important for biofilm formation and chronic infection^14^. When AlgR is phosphorylated, it controls functions related to cell attachment and initial biofilm formation, while a high excess of non-phosphorylated AlgR induces late biofilm and chronic infection traits, including alginate biosynthesis and the mucoid phenotype^12^. Several observations have also linked this system with the *P. aeruginosa* ribonucleotide reductases (RNR) network^6,12^.

Ribonucleotide reductases are the enzymes responsible for reducing the ribonucleotides (NTP) to the corresponding deoxyribonucleotides (dNTP), thereby forming the building blocks for DNA synthesis and repair^15^. There are three known RNR classes (I, II and III), and all use a free-radical-based catalysis; however, they rely on different metallo-cofactors for the initiation of the radical reduction step, and each one exhibits a different behavior towards oxygen. Class I RNR can be enzymatically active only under aerobic conditions, class II RNR is oxygen-independent and requires vitamin B_12_ for enzyme activation, and class III RNR requires strict anaerobic conditions to be active. While almost all eukaryotic organisms encode exclusively class Ia RNR, prokaryotes are known to encode more than one, in all possible combinations^16^. *P. aeruginosa* encodes all three RNR classes: class Ia (*nrdAB*), class II (*nrdJab*) and class III (*nrdDG*)^17^. Their different requirements and relationships with oxygen give them different roles throughout the *Pseudomonas* life cycle and in the biofilm structure^15,18,19^.

The RNR activity is known to be extensively regulated at both the transcriptional and post-translational levels; it is delicately modulated to keep a balanced nucleotide pool and globally regulated according to the life cycle, stress situations, and environmental conditions. However, much remains unknown about which factors allow bacteria to activate the different classes under different circumstances.

Several years ago, one of the genes found in a transcriptomics experiment to be regulated by AlgR in *P. aeruginosa* was the *nrdJ* gene (PA5497)^6^, which encodes a class II ribonucleotide reductase that plays a crucial role during biofilm formation and infection. In addition, a recent study that aimed to identify AlgZR-regulated genes using ChIP-seq showed a particular region for AlgR binding (AlgR-box) in a short DNA fragment within the intergenic region between the class I RNR operon first gene (P*nrdA*; PA1156) and the PA1157 gene^12^. All of these observations point to the existence of a relationship between the AlgZR system and the RNR network.

In this study, we aimed to uncover this relationship. We demonstrate that AlgR regulates both RNR classes I and II in a differential way, depending on its phosphorylation state. We explore how this differential regulation allows bacteria to adapt to different situations when living in a free form, during colonization of surfaces and in mucoid or non-mucoid biofilms. Finally, we unravel for the first time the molecular mechanisms behind the well-known activation of ribonucleotide reductase activity that occurs under oxidative stress.

## Results

### Ribonucleotide reduction is regulated by AlgR in *P. aeruginosa*

Previous studies have suggested a regulation by the two-component system AlgZR on class II RNR^6^. Furthermore, AlgR has been reported to bind upstream to the class Ia RNR operon^12^, facing the neighboring gene PA1157. We aimed to explore a possible regulation by AlgZR on the RNR network, and clarify if the already detected binding site regulates the RNR class Ia operon *nrdAB* or the PA1157.

We initially used plasmids carrying a transcriptional fusion of the *nrdA* (pETS134), *nrdJ* (pETS180), *nrdD* (pETS136) or PA1157 (pETS206) promoters to the green fluorescent protein (GFP). The promoter of the *algD* (and consecutive genes) operon, main responsible for alginate biosynthesis, was used here as a positive control, as it is well-known to be regulated by non-phosphorylated AlgR (pETS205)^3,14,20,21^.

**Figure 1:**
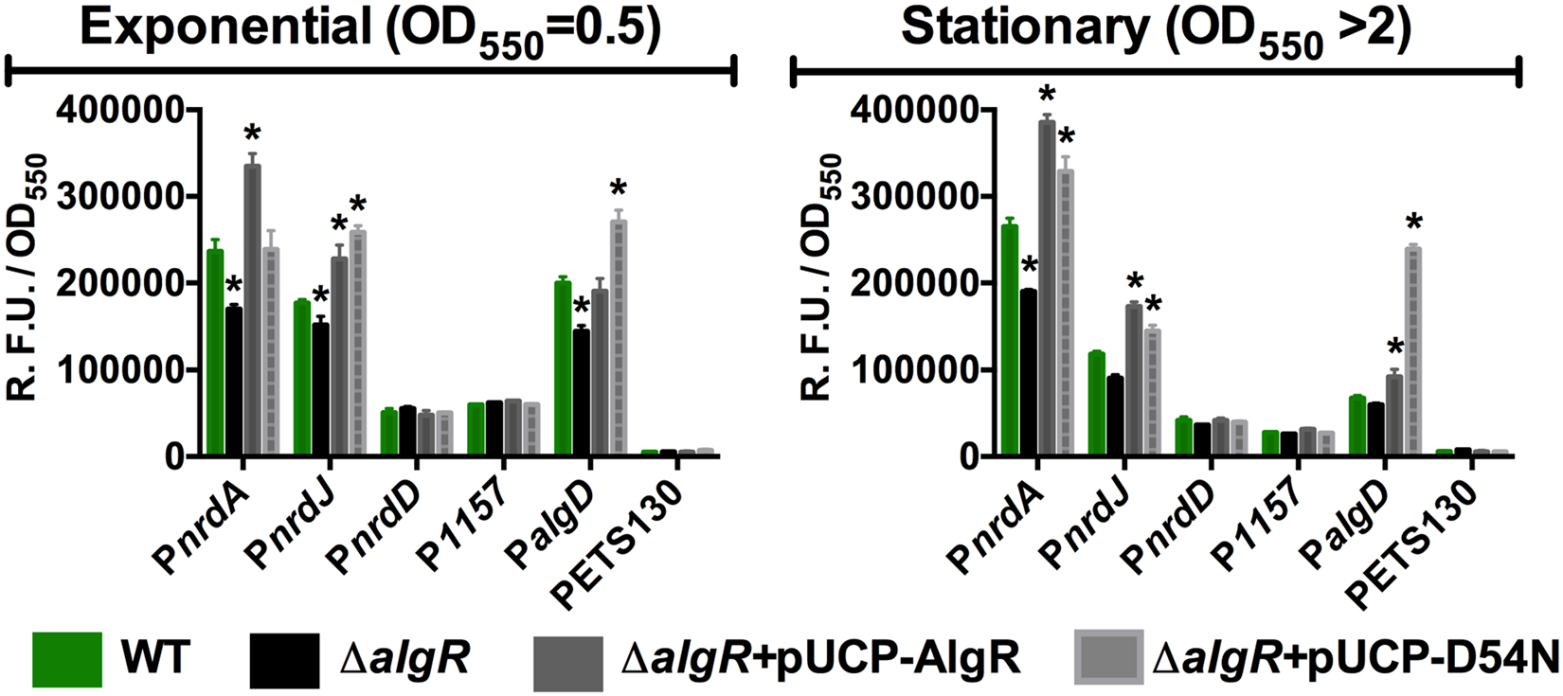
*In vivo* AlgR regulation of RNR promoters and related genes. Gene reporter assays for P*nrdA* (pETS134), P*nrdJ* (pETS180), P*nrdD* (pETS136), P_*PA1157*_ (pETS206) and P*algD* (pETS205) fused to GFP at exponential and stationary growth phases. Values are averages from at least three independent experiments, and error bars show positive standard deviation. The promoterless pETS130-GFP plasmid values are provided for comparison. Asterisks (*) indicate a statistically significant difference from the wild-type strain (*p*-value less than 0.05 in pairwise *t*-tests). Shortened names are used (see Supplementary Table S1).

As shown in Fig. 1, comparing the expression of the wild-type *P. aeruginosa* PAO1 strain with its isogenic *algR* mutant strain (∆*algR*; PW9855), the regulation of the P*algD* promoter expression by AlgR is consistent with what has been extensively reported. AlgR acts as an activator of its transcription, although it is typically almost fully inactive in the non-mucoid phenotype^3,14^.

Studying the control on the RNR operons, we detect similar positive regulation by AlgR on the class I operon (*nrdAB*) and class II operon (*nrdJab*). Complementation with a *fimS*-*algR* overexpression construct (pETS203, pUCP-AlgR) increased the expression of both promoters beyond the levels of the wild-type. The PA1157 promoter was not affected by either the absence or overexpression of the AlgR regulator. Thus, there is no evidence of the AlgR binding in the PA1156-PA1157 intergenic region regulating the PA1157 gene as previously reported^12^. Instead, it controls the adjacent *nrdAB* genes. No change was found in P*nrdD* promoter expression (pETS136), so class III RNR is not regulated by AlgZR under the studied conditions.

### The AlgZR phosphorylation switch modulates RNR regulation

As a two-component system, the biological function of the AlgR regulation is conditioned by its phosphorylation state^14^. We aimed to explore the effect of AlgR phosphorylation on the RNR genes regulation. To do so, we took advantage of the AlgR D54N mutant: it has been shown that a conserved substitution of the D54 residue of AlgR to an asparagine (AlgR D54N) abolishes its *in vitro* and *in vivo* phosphorylation by the FimS (AlgZ) kinase in response to environmental signals, while keeps protein structure apparently intact^11,20^. Hence, we used the wild-type AlgR (pUCP-AlgR) and its variant AlgRD54N (pUCP-D54N) overexpression plasmids to determine the influence of phosphorylation in regulating *nrdA* and *nrdJ* transcription (Fig. 1).

Validating our approach, AlgR D54N increases *algD* expression much more than wild-type AlgR complementation does, as AlgR needs to be non-phosphorylated to regulate positively *algD* transcription^14,22^.

Studying the RNR genes, both pUCP-AlgR and pUCP-D54N were able to increase *nrdJ* transcription levels beyond the wild-type values in a similar way, both in the exponential and stationary growth phases. Therefore, no apparent global effect of AlgR phosphorylation on RNR class II regulation could be identified under these conditions. However, the transcription of class Ia RNR (*nrdA*) showed evidence of dependence on the phosphorylation state of AlgR, presenting a higher increase in expression with phosphorylatable AlgR. Further results in other growth models demonstrated this effect (see below). The transcription of *nrdD* and PA1157 were, as expected, unresponsive to either pUCP-AlgR or pUCP-D54N overexpression.

### AlgR binds to the *nrdA* and *nrdJ* promoter regions through specific AlgR-binding boxes

**Figure 2:**
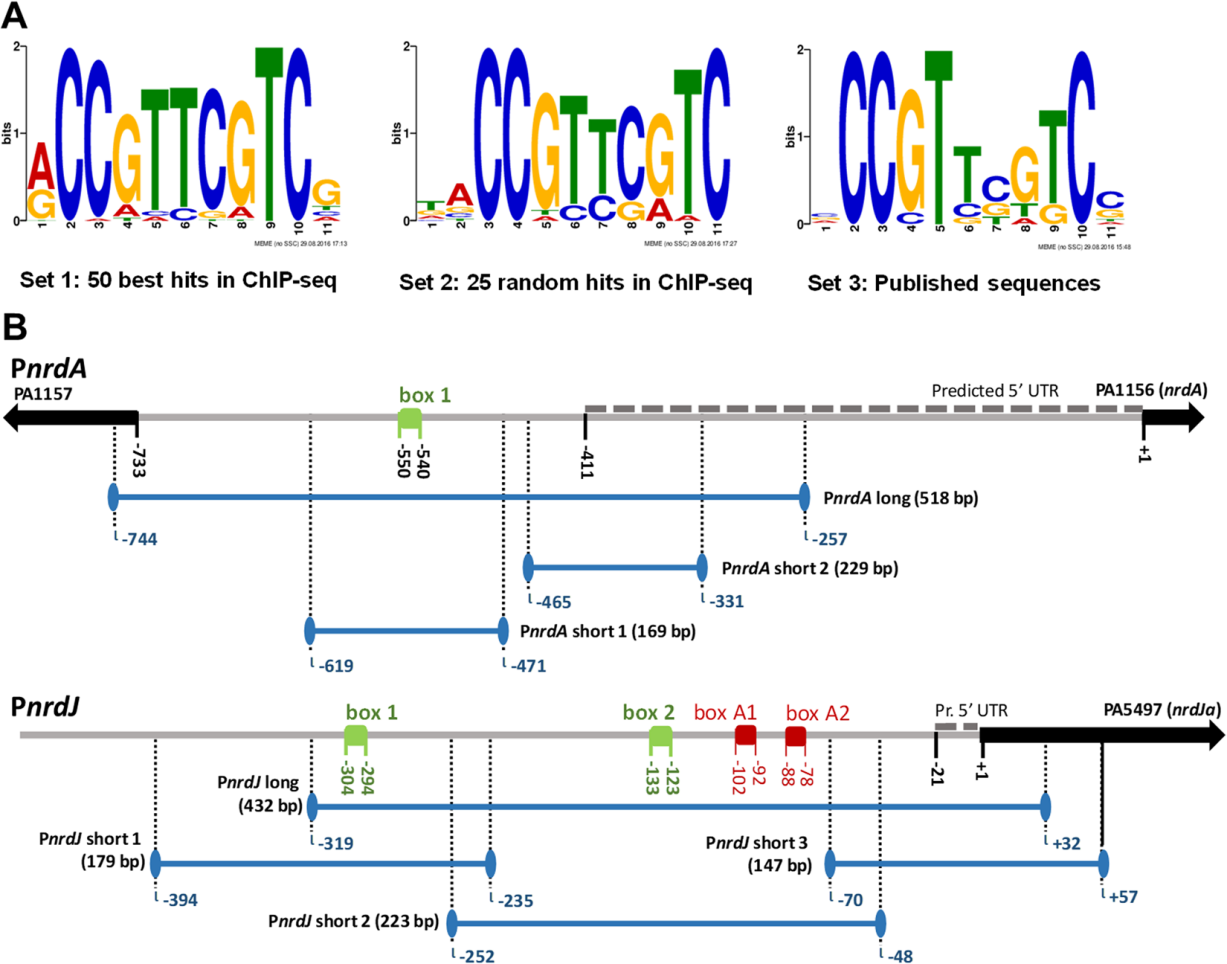
AlgR boxes in the RNR promoters. (**A**) Sequence logos for the AlgR binding box. HMM logos are generated from count matrices (see Supplementary Fig. S1) produced by FIMO using three different sets of sequences containing AlgR binding sites (see Materials and Methods). (**B**) Schematics for promoters P*nrdA* (RNR class I promoter) and P*nrdJ* (RNR class II promoter). Identified boxes are represented in green, artifact boxes identified as false positives in the bioinformatics search are represented in red. Genes are represented by arrows; gene *znuA* has been eliminated from the P*nrdJ* schematic for improved readability. An approximated prediction of the 5′UTR for the studied operons (BPROM) is shown as dashed lines. Locations are indicated in base pairs relative to the ATG translation start codon of the first gene of the corresponding operon. DNA probes used for EMSA studies are indicated by solid blue lines.

To localize the AlgR binding sites in the class Ia and II RNR promoter regions, a thorough bioinformatics search was conducted. First, to characterize the AlgR-box consensus sequence, we used MEME (MEME suite), starting from three different sources of information (see Materials and Methods), to obtain three count matrices characteristic of the AlgR binding site (Supplementary Fig. S1). A FIMO search (MEME suite) was later conducted with all three matrices. Using positive and negative control probes (see Material and Methods), we concluded that a 1e-4 p-value threshold showed no false positives and identified strong AlgR binding sites in all situations. On the other hand, a 1e-3 p-value threshold recognized all boxes with all sets but also showed up to 5 non-specific hits in the negative control. Using the three count matrices on a FIMO search of promoters P*nrdA*, P*nrdJ* and P*nrdD*, applying the 1e-4 p-value threshold, a single binding site was identified on P*nrdA* and P*nrdJ*, while no hits were retrieved from P*nrdD*. As further results showed that P*nrdJ* included more than one binding site (see below), a less stringent search was conducted for this probe, in which all hits obtained from applying a 1e-3 p-value threshold was considered. All the identified boxes are represented in Fig. 2.

**Figure 3:**
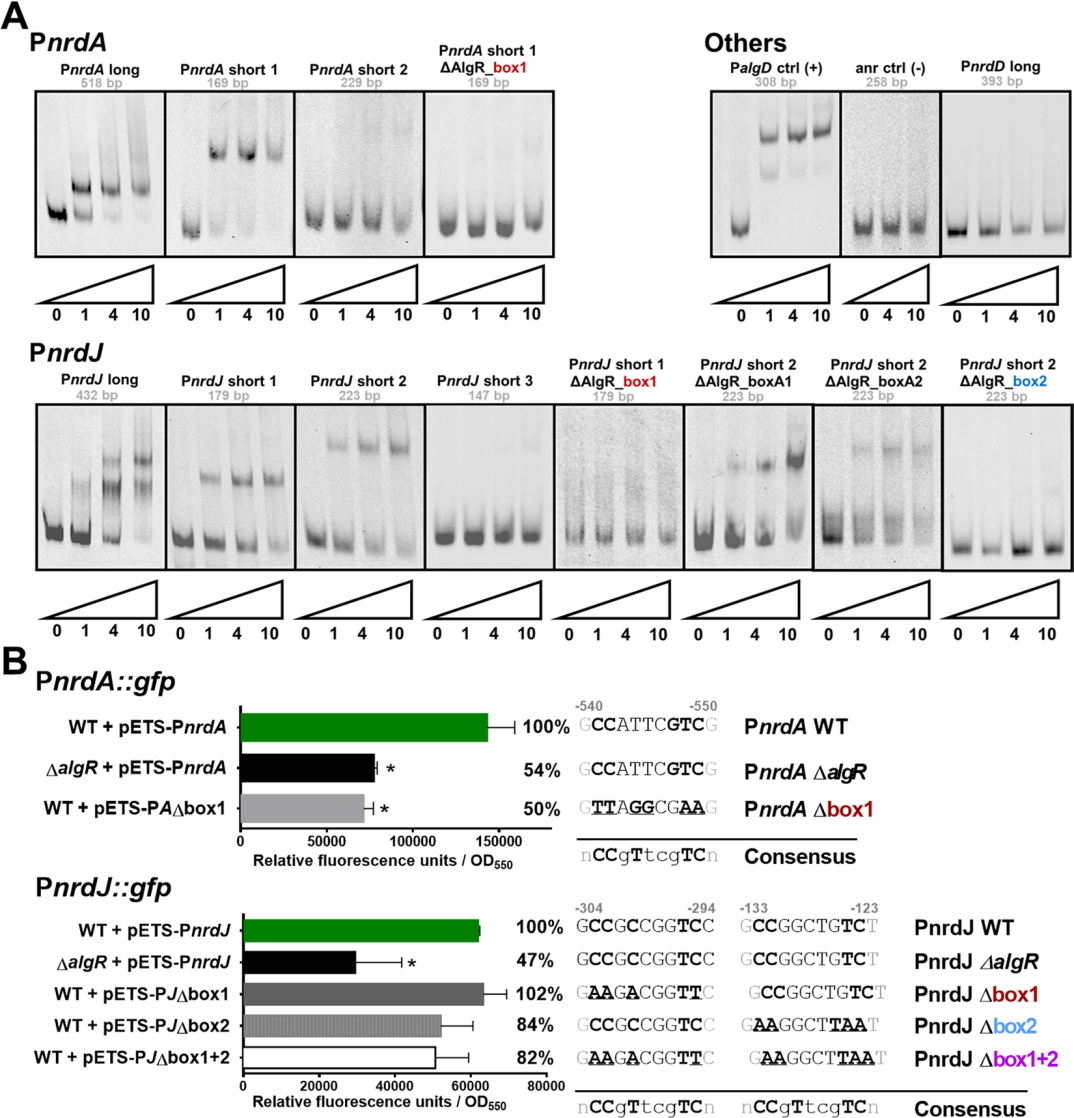
Functional study of the AlgR boxes in RNR promoters. (**A**) EMSA experiments promoters P*nrdA*, P*nrdJ*, and P*nrdD*, together with positive control (P*algD*) and negative control probes. Probe sizes are indicated below their names; numbers below the triangles represent pmol of AlgR. (**B**) Gene reporter assay for P*nrdA* and P*nrdJ*, during the early stationary phase (OD_550_ = 2.0) and under aerobic conditions. Error bars represent positive standard deviations; the asterisk indicates a statistically significant difference from the wild-type strain (*p*-value less than 0.05 in pairwise *t*-tests). The exact mutations introduced are detailed at the right of the graphic, and a simplified consensus sequence of the AlgR box is provided for comparison. The position of each box is indicated in bp (to the ATG of the first gene in the operon). Shortened names are used (see Supplementary Table S1). The images in A were cropped for clarity from the originals in Supplementary Fig. 2.

To characterize the AlgR-DNA binding activity and experimentally demonstrate AlgR binding to the identified putative boxes, we performed Electrophoretic Mobility Shift Assays (EMSA). Initially, long DNA probes spanning all the predicted promoter regions for class I (P*nrdA*), class II (P*nrdJ*) and class III (P*nrdD*) were analyzed, with the corresponding positive control (a band of the P*algD* promoter including its two strong binding sites) and a negative control. The P*algD* band showed, as expected, evidence of two strong binding events. We also identified one binding activity in the P*nrdA* promoter and two binding events in the P*nrdJ* promoter, while no evidence of an AlgR-DNA interaction was found for P*nrdD* (Fig. 3A).

To localize more precisely the AlgR binding locations, we segmented them into smaller DNA probes (Fig. 2). As seen in Fig. 3A, we established that binding activity was localized to one location in probe P*nrdA* short 1, as well as in one location in P*nrdJ* short 1 and another in P*nrdJ* short 2. We then proceeded with putative AlgR box mutagenesis, determining as final binding sites the ones now labeled as P*nrdA* box 1, P*nrdJ* box 1 and P*nrdJ* box 2, whose mutagenesis abolished DNA shifts. The other boxes we proposed in P*nrdJ* short 2 are considered artifacts of the bioinformatic search. P*nrdA* box 1 colocalizes with the DNA fragment enriched by AlgR-precipitation in ChIP-seq.^12^. Therefore, the identified boxes confirmed the presence of the previously described AlgR binding site in the intragenic region between the *nrdA* and PA1157 genes^12^ and also included previously unreported putative binding sites in the class II RNR promoter region.

The *in vivo* effect of the described boxes was first assessed under liquid culture conditions by using promoter-GFP fusions in gene reporter assays (Fig. 3B). In P*nrdA* class I RNR, we determined that the identified AlgR box is fully responsible for the AlgR regulation of this promoter, as mutation of this box resembles the effect of mutating the *algR* gene. The effect of the boxes identified in P*nrdJ* class II RNR is complex; even though it was demonstrated that mutating box 1 abolished AlgR binding in the immediate region, this mutation had no significant effect on P*nrdJ* expression in liquid cultures. Mutating box 2 or both boxes reduced the P*nrdJ* expression, but not to the levels seen in a ∆*algR* mutant strain. The effect of P*nrdJ* AlgR boxes is further studied below, under different conditions.

Finally, when comparing the identified AlgR boxes with those previously known, we realized that P*nrdA* box 1 is more similar to those described as “strong binders,” while P*nrdJ* boxes resemble the so-called “weak-binders” (Supplementary Fig. S3)^14^. Specifically, there is one cytosine in position 7 present in all strong binders that is absent in all weak binders. A comparative EMSA with a wide array of protein concentrations shows that, as expected, binding in P*nrdA* and P*algD* requires smaller quantities of protein and results in sharper, more stable bands. Binding in P*nrdJ* requires higher protein levels for full occupation of both boxes and forms blurrier bands, indicative of a more unstable complex.

### AlgR binding on RNR promoters alters the DNA structure

It has been reported that AlgR control, usually performed through binding hundreds of base pairs upstream of the basal promoter, often implies DNA bending^14^. In the regulation of the promoter of the *algD* operon, the best-known AlgR regulatory process, a wide DNA loop is formed integrating the actions of AlgR on its three binding locations (strong sites RB1/RB2, weak site RB3) and other proteins^14,21^. However, there is no published visual evidence of this process, and no studies have demonstrated if AlgR can alter the DNA structure by itself, in the absence of other factors.

**Figure 4:**
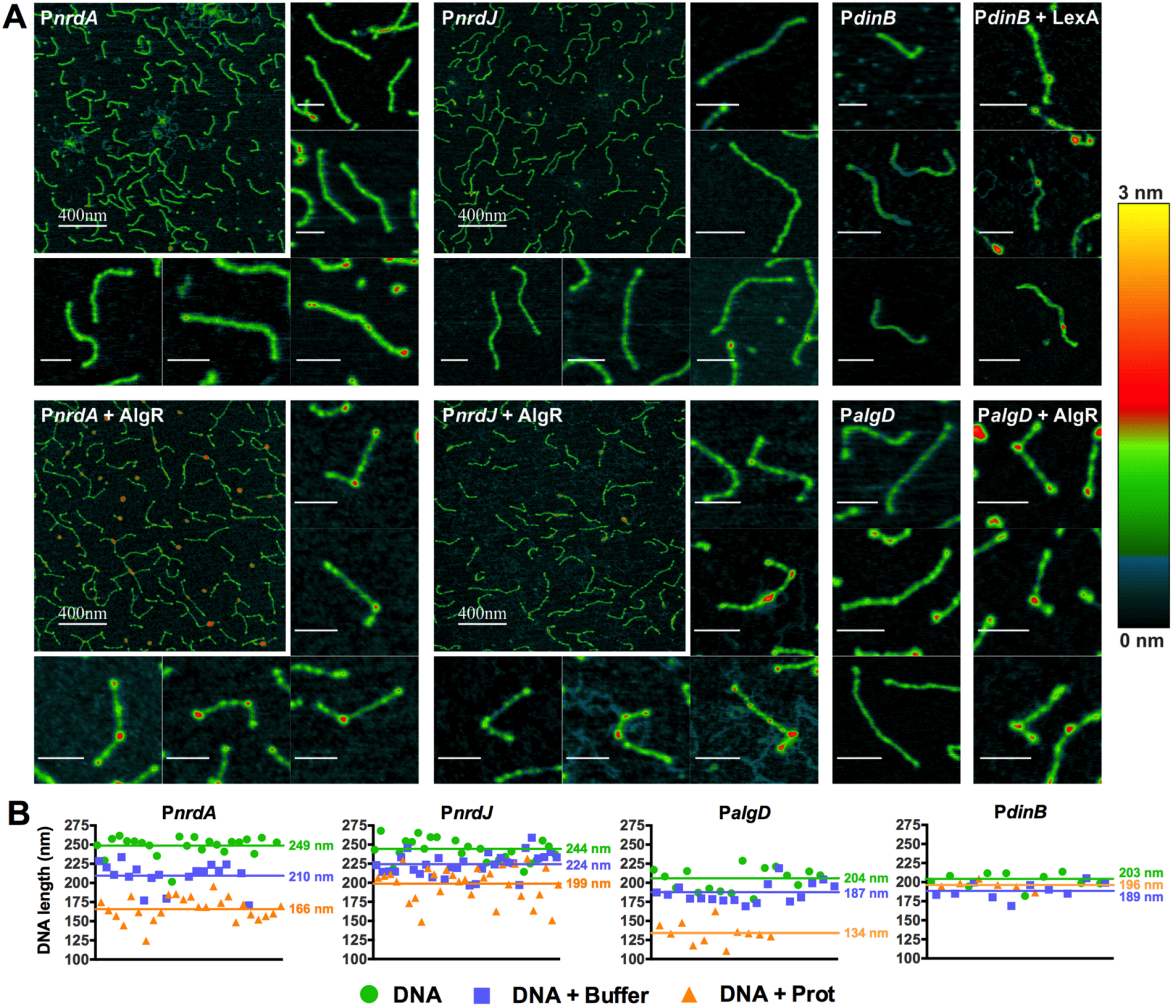
Atomic force microscopy images of DNA and DNA-protein complexes. (**A**) AFM images of DNA molecules or DNA-protein complexes, taken on mica under ambient conditions, are shown for P*nrdA*, P*nrdJ*, P*algD* and P*dinB* promoters. Small images depict single DNA probes; scale bars without numbers above represent 80 nm. For the *PnrdA* and P*nrdJ* promoters, a general image at a higher scale is also shown. Colors represent the height of the structures, according to the scale at the right. (**B**) comparison of the apparent length of randomly selected units of all DNA probes when, before drying, they were in water (images not shown) or in binding buffer, as well as when complexed with AlgR.

To explore the physical effect of AlgR binding on RNR promoters, we observed previously formed DNA*-*AlgR complexes, compared to free DNA probes, using Atomic Force Microscopy (AFM) (Fig. 4). AlgR binding can be observed on both P*nrdA* and P*nrdJ* probes (yellow/red spot), although several series of images showed that the P*nrdA* complex was easier to obtain and more stable. One single binding site is observed in the P*nrdA* promoter, while the P*nrdJ*-AlgR complexes show bindings in two locations. There is also a binding event on two sites in the P*algD* probe, containing its two strong boxes RB1/RB2.

Despite the possible artifact introduced by the natural positioning of the DNA probes on the mica surface, we can observe that AlgR binding colocalizes with a remarkable DNA bending event. To explore the nature of these bindings, they were compared to the very well-known binding of the LexA repressor to the damage-inducible DNA polymerase IV (*dinB)* promoter region^23^. No bending is observed due to LexA binding on P*dinB* (Fig. 4A).

Moreover, we determined the apparent length of the DNA fragments in the AFM images (Fig. 4B), observing that although it was already reduced when the DNA was in the protein binding buffer rather than in water prior to drying, it got reduced to a greater extent when AlgR protein was bound. This effect is quite apparent in P*nrdA* and P*algD* probes and is also detectable in P*nrdJ*, whereas no evidence of it is found due to LexA-P*dinB* binding.

### AlgR regulation during surface colonization reveals a complex mechanism behind RNR transcription fine tuning

The AlgZR system is required for fimbrial biogenesis^11^ and rhamnolipid formation^24^, both activities of the utmost importance for surface colonization and colony and biofilm formation^25,26^. We, therefore, considered it necessary to explore the AlgR regulation of *nrd* genes during surface growth. In our surface colonization experiments, different strains harboring promoter::*gfp* fusion plasmids were grown on agar plates for 36 h, and fluorescence was determined at 3-h intervals during all growth. This model is also useful for exploring the AlgR action on *nrd* genes in the mucoid phenotype, using the *P. aeruginosa* PAOMA (∆*mucA*) strain, which forms very characteristic mucoid colonies.

**Figure 5:**
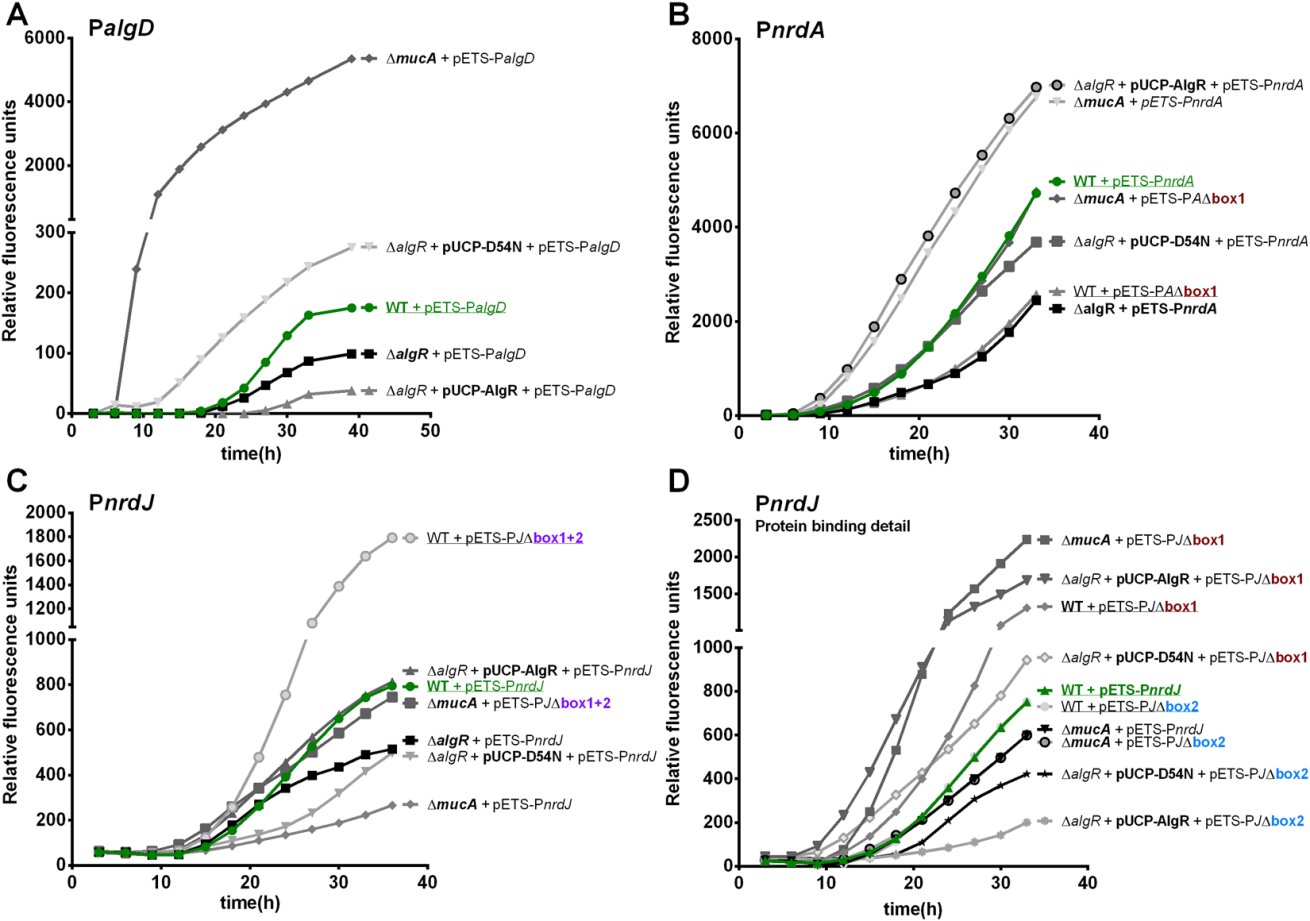
AlgR regulation of RNR promoters during surface colonization. GFP-based gene reporter assays for P*algD* (pETS205), (**A**) P*nrdA* (pETS134), (**B**) and P*nrdJ* (pETS180), (**C**) promoters fused to GFP, during surface colonization. GFP fluorescence is measured at different times of incubation during colony formation and presented as relative fluorescence units. Mucoid strains (PAOMA, PAO Δ*mucA*) are included. A fourth panel (**D**) shows further experiments with P*nrdJ* AlgR boxes to study the fine regulation performed at this level. For improved readability, shortened names are used (see Supplementary Table S1), the key features of each strain are highlighted in bold, wild-type strains are underlined and mutant boxes are color-coded.

We first analyzed the regulation of the P*algD* promoter (Fig. 5A). The basal level of P*algD* expression in a non-mucoid phenotype is very low, although it can be seen that the ∆*algR* deletion reduces its expression and that it can be complemented by the non-phosphorylatable AlgR D54N protein, whereas the wild-type protein does not complement (or even slightly inhibits) P*algD* transcription. In the ∆*mucA* strain, the great increase in non-phosphorylated AlgR levels causes a very significant increase in P*algD* transcription (>6000 RFU). All results agree with our previous observations and published data^3,14,27^, serving as a control for this technique.

For the class I RNR P*nrdA* promoter (Fig. 5B), the results confirm what was observed in the liquid cultures, although they are more evident under these conditions: mutating the *algR* gene causes a clear reduction in *nrdAB* transcription, and mutating the AlgR-box in the promoter mimics this effect. Complementation with AlgR wild-type protein over-activates the promoter whereas D54N is not able to fully complement the mutation, demonstrating that AlgR phosphorylation is required for the induction of class I RNR.

The results are more complex for the class II RNR P*nrdJ* promoter (Fig. 5C). As previously described (Fig. 1), mutating the *algR* gene causes a reduction in P*nrdJ* transcription, much evident than in liquid cultures, which can be complemented by introducing additional copies of *algR* (pUCP-AlgR). The overexpression of wild-type AlgR protein complements the mutation, while, in this model, AlgR D54N overexpression causes a reduction of the operon transcription. This first evidence that accumulation of AlgR can inhibit P*nrdJ* transcription is supported by the fact that, unlike for P*nrdA*, the activity of the promoter is severely reduced in the mucoid phenotype (∆*mucA*). Additionally, mutation of both AlgR boxes in the P*nrdJ* promoter causes not a reduction, but a significant increase in the transcription of the promoter, indicating a more complicated underlying mechanism.

To explore the independent action of the AlgR boxes, we performed several colony formation experiments with single-box mutants (Fig. 5D). Both boxes display very different behaviors. Mutating box 1 increases the expression of the promoter, therefore suggesting that AlgR is inhibiting P*nrdJ* transcription by binding to box 1. Eliminating box 1 can switch the effect of the ∆*mucA* background from a significant reduction to a large increase in transcription, suggesting that the previously observed inhibition of class II RNR transcription in the mucoid phenotype happens through AlgR binding in the AlgR box 1. The effect of mutating box 2 is not detectable in a wild-type background, but its mutation switches the effect of the AlgR-overexpression strain from an increase (even higher with phosphorylatable AlgR) to a dramatic decrease in P*nrdJ* transcription. Box 2 is therefore proposed to be implicated in P*nrdJ* activation in response to AlgR phosphorylation, in competition with the action of box 1, which would be involved in P*nrdJ* inhibition in the mucoid phenotype. The implications of this dual mechanism on stress conditions and the mucoid phenotype are further discussed below (see Discussion).

### The AlgR regulation mechanism is reproduced in mucoid and non-mucoid biofilms

**Figure 6:**
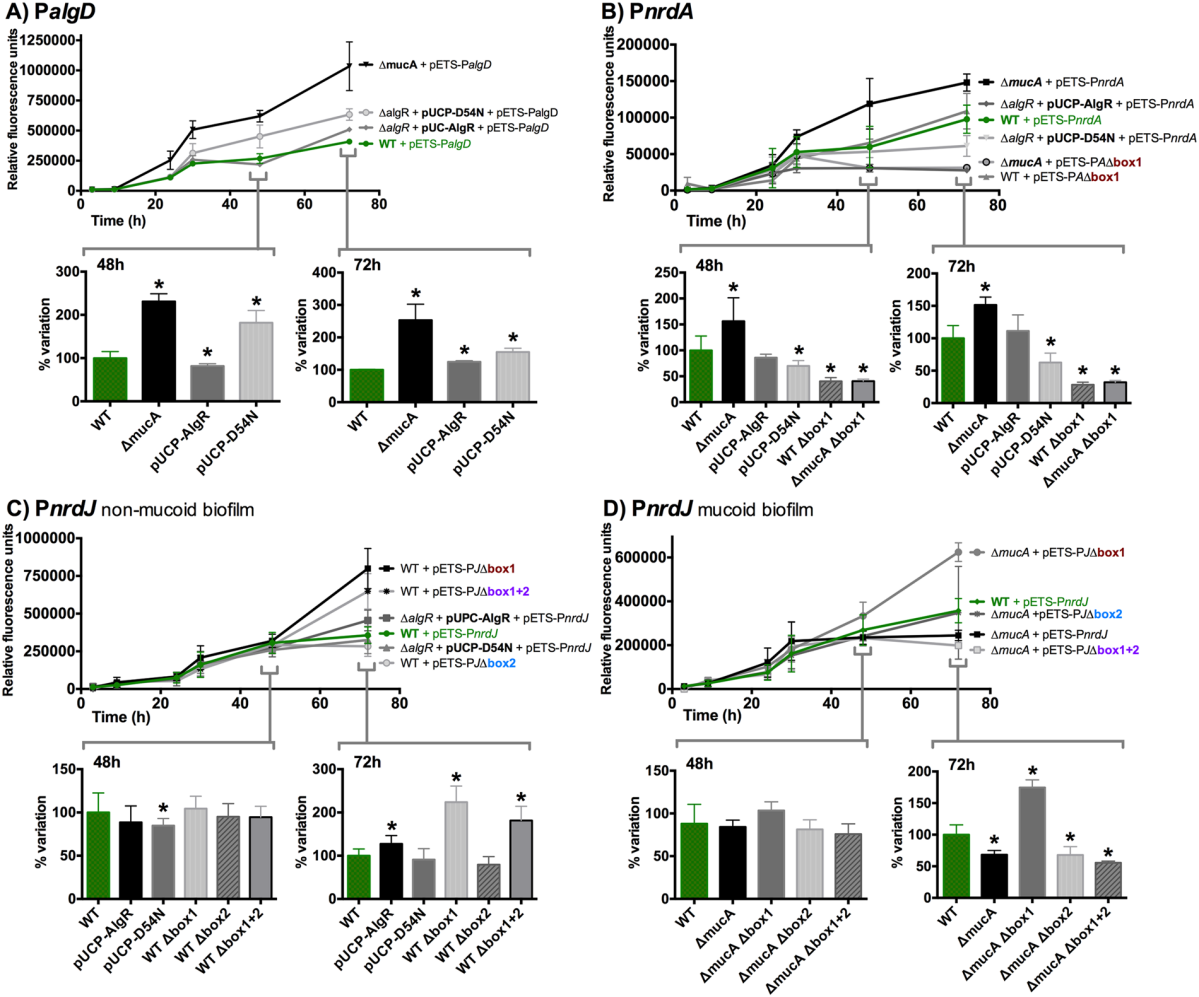
AlgR regulation of RNR promoters in mucoid and non-mucoid biofilms. Gene reporter assay at different time points during static biofilm formation for P*algD* (pETS205), (**A**), P*nrdA* (pETS134), (**B**) and P*nrdJ* (pETS180), **(C** and **D**). The values shown are the means of three independent experiments in 8 wells; error bars indicate positive and negative standard deviation. Shortened names are used (see Supplementary Table S1). For 48 h and 72 h, results are depicted as bar graphs; error bars show positive standard deviation, and the asterisk indicates a statistically significant difference from the wild-type strain (*p*-value less than 0.05 in pairwise *t*-tests).

Our group recently demonstrated the importance of class II RNR (*nrdJab*) during *P. aeruginosa* biofilm formation and its transcriptional activation by anaerobic regulators under this condition^19^. However, the regulators involved in modulating RNR transcription in the biofilm are still unknown. The AlgZR system has been extensively associated with different aspects of biofilm formation, and here we have demonstrated that it controls the *nrd* genes, which are also differentially regulated in the mucoid phenotype. Therefore, we decided to explore the modulation of class I and II RNR expression by AlgZR in mucoid and non-mucoid biofilms. In Fig. 6, we determined the *nrdA* and *nrdJ* expression, together with the *algD* expression as a control, during biofilm formation. Measurements were taken at different time intervals during growth (from 3 h to 72 h). The un-complemented PW9855 (∆*algR* mutant) strain could not be used, as it presents severely impaired biofilm formation capabilities.

As in our previous experiments in colonies, AlgR functions in the biofilm as an activator of P*algD* transcription, where it is more responsive to non-phosphorylatable AlgR D54N than to wild-type AlgR, and shows a high induction in the mucoid *P. aeruginosa* PAOMA (∆*mucA*) strain (Fig. 6A).

Class I RNR transcription (Fig. 6B) is induced in the mucoid biofilm at a very early stage in its formation. P*nrdA* induction occurs only due to phosphorylated AlgR overexpression and not with its non-phosphorylatable counterpart AlgR D54N (although the effect of AlgR overexpression does not go beyond complementing the mutation). The AlgZR regulation appears to be responsible for the effect in the mucoid biofilm, as mutating AlgR box 1 eliminates this induction, as well as in the non-mucoid variant.

Finally, the complex regulation of class II RNR is also reproduced in biofilm formation conditions (Fig. 6C and D). The changes in transcription can be more easily detected in mature biofilms (72 h), while younger biofilms show almost no evidence of regulation. In a mature mucoid biofilm, there is a clear reduction in P*nrdJ* expression, which can be restored with the mutation of AlgR box 1. Mutating this box causes a general increase in class II transcription while mutating box 2 causes a reduction. The double mutation causes an opposite effect in both the regular mature biofilm and in the mucoid biofilm. The differential action of AlgR box 1 and AlgR box 2 is therefore demonstrated, and it is related to both AlgR-mediated induction of class II RNR in non-mucoid biofilms and AlgR-mediated repression of class II RNR in the mucoid biofilm.

### Ribonucleotide reductase induction under oxidative stress acts through AlgR regulation

There are several reports which describe that RNR activity is strongly activated under oxidative stress conditions by increasing *nrd* gene transcription through an unknown molecular mechanism^15,28,29^. Here, we explore the ability of AlgR to sense oxidative stress and accordingly regulate RNR gene expression.

**Figure 7:**
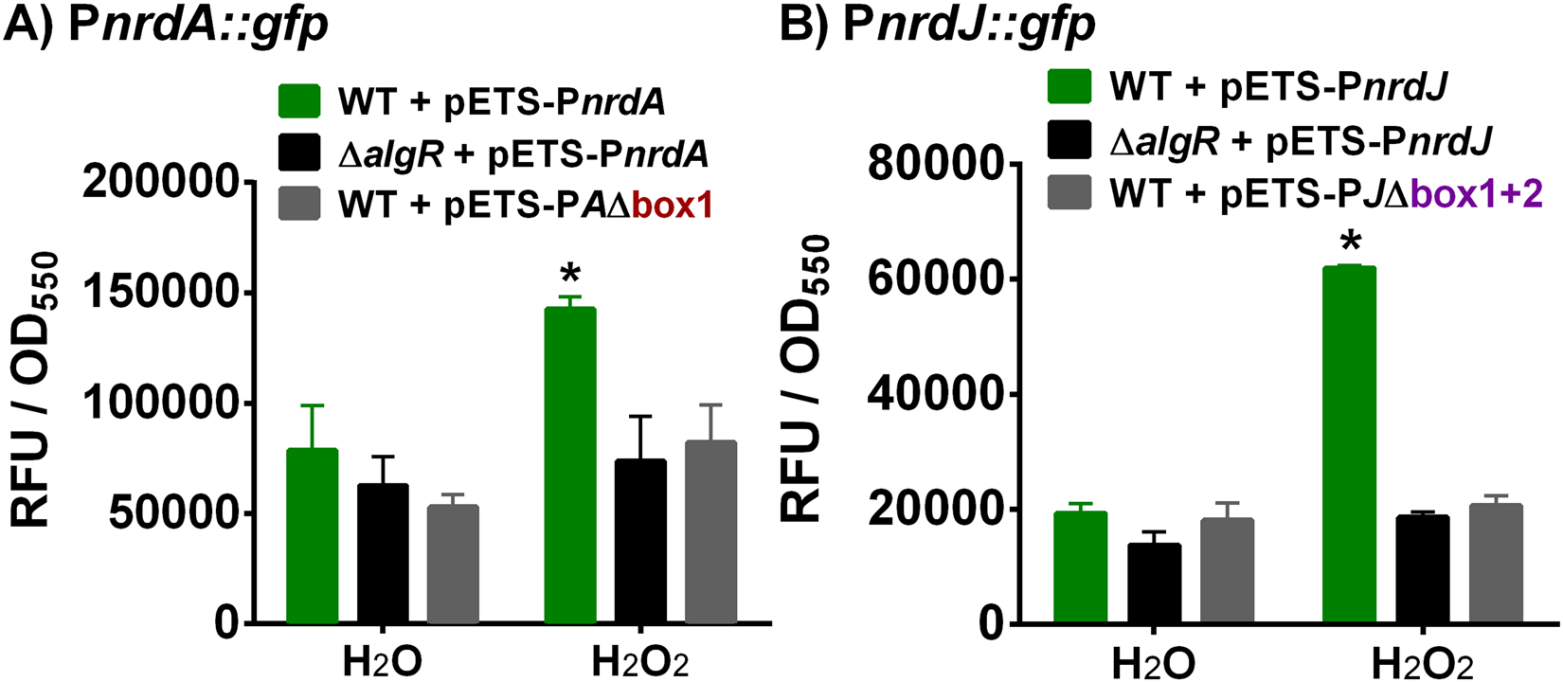
AlgR regulation of RNR during oxidative stress. Gene reporter assays for the P*nrdA* and P*nrdJ* promoters fused to GFP. All strains were grown to OD_550_ = 0.5 and then subjected to 30 minutes of incubation with a stressing agent (1.0 mM H_2_O_2_) or control (equivalent volume of water). Values are averages from three independent experiments, and error bars show positive standard deviation. Asterisks (*) indicate statistically significant difference from the untreated wild-type strain (*p*-value less than 0.05 in pairwise *t*-tests). Shortened names are used (see Supplementary Table S1).

As hypothesized, class I (*nrdA*) and II (*nrdJ*) RNR respond to oxidative stress (induced by hydrogen peroxide treatment) by significantly increasing their transcription (Fig. 7). Surprisingly, this response to oxidative stress is entirely abolished if the *algR* gene is inactivated (∆*algR* mutant strain). Introducing mutations in the identified AlgR binding regions of the *nrdA* and *nrdJ* promoter regions mimics the effect of the *algR* isogenic mutant, rendering them unable to respond to oxidative stress.

These results indicate, for the first time, that the well-described activation of the *nrd* genes by oxidative stress occurs through the action of the AlgZR two-component system.

## Discussion

As an essential activity for the life of any cell, ribonucleotide reduction is always thoroughly regulated. In bacteria, where different RNR classes could be present and required for changing situations, the activation and inactivation of the several classes add another layer of complexity.

Several pieces of this regulatory puzzle are already known. In *P. aeruginosa*, apart from class Ia (whose transcription has been mainly studied in *E. coli*), class II is known to be especially important in biofilms and positively regulated by the anaerobic system Anr/Dnr, although we proposed the action of other biofilm-related factors^19^. Class III is highly activated under strictly anaerobic conditions by still unknown regulators. Moreover, the global regulator NrdR, which negatively regulates all RNR expression in almost all bacterial species^15^, is also present in the *P. aeruginosa* network^30^. However, despite all known information, there are yet many missing pieces. RNR activity is modulated under oxidative stress conditions^28,29^ and specific environmental conditions through unknown factors. The data linking the AlgZR two-component system to the RNR network^6,12^ could reveal another piece of this complex regulation.

For our bioinformatics analysis, we took advantage of published information regarding DNA sequences that bind AlgR^12^, but we realized that these data accounted only for high-affinity binders. Therefore, we also used several published sequences^14^, including weak binders, to produce a more relaxed search pattern (Supplementary Fig. S1). We identified putative binding sites in the P*nrdA* and P*nrdJ* promoters (Fig. 2) that were experimentally demonstrated (Fig. 3A); the absence of boxes in P*nrdD* suggests that class III RNR is not regulated by AlgZR. Contrary to the well-known *algD* promoter, which contains 3 AlgR-boxes (RB1, RB2, and RB3)^3,31^, one unique AlgR-box was identified in the P*nrdA* promoter, and two were identified in the P*nrdJ* promoter. Although members of the AgrA family such as AlgR usually bind to direct repeats of their binding sequence^14^, it is known that other genes regulated by AlgR contain different numbers of boxes in their promoters (*algD*, 3 boxes; fimU, 2 boxes; *hcnA*, 1 box; *rhlA*, 1 box; *rhlI*, 1 box). The distance from these boxes to their predicted transcription start sites (from 100 bp to 300 bp; Fig. 2) suggests that DNA bending will be necessary to interact with the transcription machinery. A deeper analysis of their sequences also reveals that the box in the P*nrdA* promoter is that of a strong binder, while P*nrdJ* boxes resemble that of known weak binders (Supplementary Fig. S3B). The cytosine (C) in position 7 is present only in strong binding sequences; this difference can be used to conduct new bioinformatics searches specifically geared towards AlgR weak-binding sequences.

Consequently, the results of the AFM imaging (Fig. 4) of the DNA-protein complexes confirmed that binding of AlgR to the RNR promoters causes bending of the DNA; this explains how binding sites that are so far away from the transcription start site can interfere with transcription. Although AlgR-mediated bending has been proposed many times, to our knowledge, this is the first time that it has been experimentally demonstrated. The so-formed loops suggest interactions with other factors, such as the Anr/Dnr system, which also regulates class II RNR.

In studying the AlgR *in vivo* regulation of the RNR network, we used different models of growth to analyze its effects under different metabolic conditions: liquid cultures (Figs 1 and 3B), a model for surface colonization (Fig. 5), and a model for biofilm formation (Fig. 6). Wild-type, Δ*algR* and AlgR/AlgR D54N complementation strains were used on all models; these strains grow at comparable rates, although the complementation strains present a slight growth reduction in early exponential phase (data not shown). The P*algD* promoter was used as a control, demonstrating that all methods are suitable for studying the effects of the AlgZR system; in all models, it acts as an activator of P*algD*, whose transcription is activated by non-phosphorylated AlgR. As expected, under some circumstances its basal expression is not sufficient to observe an effect when mutating the gene, and regulation becomes apparent only when over-activating it. Using these methods, we identified a clear control by the AlgZR system of class I and class II RNR, while class III RNR demonstrated no evidence of regulation. This was also assayed under anaerobic conditions (in liquid cultures and surface colonization models; the biofilm forms its own anaerobic areas) but no differences worth highlighting appear (data not shown). The bioinformatics search identified binding sites only where regulation was later demonstrated.

For class I RNR regulation, we determined that AlgR is activating P*nrdA* transcription (Fig. 1). The identified box, which correlates with the DNA fragment recovered in prior ChIP-seq experiments^12^, despite facing PA1157, is regulating the *nrdAB* operon (Figs 1 and 3). Other boxes with the same orientation have been described, such as the RB3 site on *algD* ^10^. The functionality of the box can be demonstrated *in vivo* (Fig. 3B), and the effects of mutating the box or the gene are reproducible and even more evident during surface colonization (Fig. 5B) or biofilm formation (Fig. 6B). We used the AlgR D54N mutant to determine the involvement of AlgR phosphorylation in *nrd* regulation^11,14^, determining that phosphorylatable AlgR is a better inducer of class I RNR expression in all models. It is known that several stress conditions, such as oxidative stress, induce *nrd* transcription^28,29^ and can also activate the two-component AlgZR system, inducing genes for cell attachment and biofilm formation^6,32^. We hypothesized that the kinase FimS could respond to stress or stress-derived signals to activate the phosphorylation of AlgR. This would give significance to P*nrdA* control by AlgR, which would be activating it in response to stress conditions. Additionally, our surface colonization and static biofilm models (Figs 5B and 6B) determined that class I RNR is induced in the mucoid phenotype and that this happens, at least partially, through AlgZR control. As non-phosphorylated AlgR, the form that is mostly predominant on the mucoid phenotype, has demonstrated less capacity to induce P*nrdA*, but is still capable to complement at least partially the Δ*algR* mutation, it is possible that this induction happens as a collateral effect of the great increase in AlgR levels.

Concerning class II RNR regulation, initial experiments suggested that it was also activated by AlgZR (Fig. 1); however, the mutation of the identified boxes quickly suggested a more complex mechanism (Fig. 3B). Surprisingly, although mutating the gene causes a reduction in P*nrdJ* expression, mutating both boxes in a wild-type background caused an increase in biofilm and colony formation models (Figs 5C and 6C); this suggests that AlgR could be acting both directly and indirectly on class II RNR expression. A more detailed mutagenesis of the boxes (Figs 5D and 6C,D) revealed that mutating box 2 causes a slight reduction of class II expression while mutating box 1 causes a clear induction in biofilms or colonies, an effect that was not seen in liquid cultures. The simplest explanation is that the positive regulation by AlgZR occurs through binding to AlgR box 2, whereas box 1 is responsible for inhibition under some circumstances. By overexpressing AlgR and AlgR D54N, we realized that the wild-type AlgR protein can complement the mutation, while AlgR D54N causes a clear inhibition in colonies and biofilms (Figs 5C,D and 6C). However, mutating AlgR box 2 can immediately switch the effect of AlgR overexpression from an induction to a strong repression (Fig. 5D). We, therefore, deduce that under some circumstances the AlgR protein can bind to box 1 to inhibit class II RNR. In the mucoid phenotype, we can see a surprisingly strong reduction of class II expression in both colonies and biofilms, but this changes to an even higher induction when mutating box 1 (Figs 5C and 6D). We propose that box 2 could be responsible for increasing P*nrdJ* expression under some stress conditions. Meanwhile, box 1 could be inhibiting class II RNR expression in the mucoid phenotype (likely in favor of class III RNR activity, but further experiments will be needed to determine this).

In light of the differences observed with AlgR phosphorylation, which must be dependent on an external signal, we tested the effect of oxidative stress; this condition is reported to dramatically induce RNR transcription through unknown mechanisms^28,29^. Surprisingly, we demonstrated that despite being in the exponential phase, where AlgR regulation is not normally very prominent, eliminating the AlgR system caused the RNR network to be insensitive to stress (Fig. 7). This is, to our knowledge, the first description of a molecular link between oxidative stress conditions and RNR expression.

**Figure 8:**
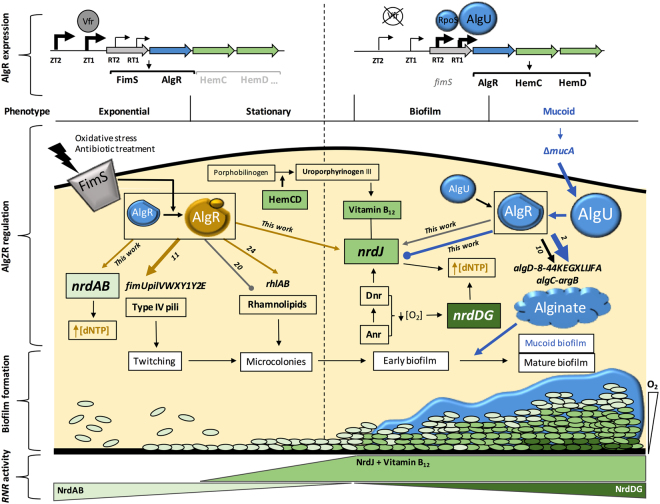
Model of AlgZR regulation of ribonucleotide reduction. Schematic representation of the AlgZR regulation of ribonucleotide reduction, in the context of other AlgZR-mediated regulation events. Arrows represent positive regulation, while lines with a bullet point represent negative regulation. Events characteristic of phosphorylated AlgR are highlighted in yellow, and events happening mostly in the mucoid phenotype are highlighted in blue. The representation is not exhaustive, and some events are eliminated for the sake of clarity. The source of information for any AlgR regulative event is indicated beside the corresponding line.

Based on these results, we suggest a model for *nrd* regulation by the AlgZR system (Fig. 8). In this model, on the one hand, class I and class II RNR are being activated by AlgR under planktonic or early colonies/biofilms, responding to AlgR phosphorylation under stress. On the other hand, in the mucoid biofilm, the high accumulation of non-phosphorylated AlgR would cause an inhibition of class II RNR through binding on box 1.

Examining this throughout all biofilm life cycle, when planktonic cells suffer the presence of a stress condition (oxidative stress, antibiotic treatment, etc.) it activates AlgR^13,14,33^. Under these conditions, the *algR* gene is expressed from promoters ZT1 (further activated by Vfr) and ZT2 (constitutive) as *fimS-algR* ^33^, a combination that favors phosphorylation. AlgR will induce the *nrdAB* and *nrdJ* genes, the operon of *fimUpilVWXY1Y2E* (Type IV pili) for the adhesion to surfaces^34^, and the *rhlAB* for quorum sensing cell communication and microcolony formation^24^. In the absence of these genes, a biofilm is not formed, and cells are more sensitive to stress conditions or antibiotic treatment^13,25^. When *P. aeruginosa* is attached to the surface, cells grow as microcolonies, which can slowly evolve to a biofilm phenotype. In biofilm conditions, a further induction of the RT1 and RT2 promoters in the *algR* operon starts to appear, controlled by AlgU and RpoS, and *algR* is therefore also expressed as *algR-hemCD* (avoiding the *fimS* gene and so being non-phosphorylated). Then, AlgR favors the induction of genes such as the *algD* and *algC* operons for the synthesis of alginate^33^ and *hemC* and *hemD* for the synthesis of the heme group (as well as allowing the formation of uroporphyrinogen III, a precursor of vitamin B_12_^35^ which is the essential cofactor for NrdJ activity). NrdJ is also induced by the Dnr transcriptional factor^19^. When biofilm becomes mature, fully anaerobic conditions appear^36,37^, inducing both *nrdJab* and *nrdDG* ^19^. It has been reported that many genes regulated by AlgR are also controlled by anaerobic transcriptional factors such as Anr or Dnr (*arcDABC*, *ccoP2*, *hcnB*, *oprG*, *hemN or nrdJ*)^6,14,19,38–42^. We observed a significant decrease when inhibiting AlgR and Dnr binding and a gradual reduction of P*nrdJ* expression when mutating both systems (Supplementary Fig. S4). If selected mutations degenerate the biofilm into a mucoid phenotype, the full release of AlgU from MucA causes a dramatic induction of RT1 and RT2 promoters in P*algR*, highly increasing the cellular levels of non-phosphorylated AlgR, and in turn decreasing the expression of NrdJ by binding to P*nrdJ* AlgR-box 1. This favors class III RNR, which is better-suited for full anaerobiosis and does not require vitamin B_12_.

Several pieces of information remain unknown. The fact that mutating the *algR* gene has a different effect than mutating both AlgR-boxes on P*nrdJ* shows us that there could be more regulation events that have not yet been described. Besides, the differences observed between surface colonization and biofilm formation conditions suggest interactions with other factors. However, we believe that these new results bring us closer to understanding the regulation of complex RNR networks such as that of *P. aeruginosa*, as well as how it adapts to environmental conditions and evolves throughout the biofilm life cycle. The link between oxidative stress, the AlgZR system, and RNR regulation provides, for the first time, a molecular explanation for this effect. In most bacterial species, there are no described analogs of the AlgZR system, but there are AgrA family two-component systems which could be candidates; further experiments will be conducted to evaluate whether these results extend to other bacterial species.

## Methods

### Bacterial strains, plasmids and growth conditions

Different *Pseudomonas aeruginosa* and *Escherichia coli* strains were used, as listed in Supplementary Table S1. Bacteria were routinely grown in LB medium (Scharlab, Spain) at 37 °C; when needed, antibiotics were added at the following concentrations: 100 µg ml^−1^ gentamicin, 40 µg ml^−1^ tetracycline, or 500 µg ml^−1^ carbenicillin for *P. aeruginosa* and 30 µg ml^−1^ chloramphenicol, 10 µg ml^−1^ gentamicin, 50 µg ml^−1^ ampicillin and 50 µg ml^−1^ kanamycin for *E. coli*. Anaerobic growth was performed in LB medium containing 10 g/l KNO_3_ in screw-cap tubes (Hungate Tubes) that were filled to the top with N2.

### DNA manipulation and plasmid construction

Molecular biology enzymes and kits were purchased from Fermentas (ThermoFisher) and used according to the manufacturer’s instructions. DNA amplification was performed by PCR using DreamTaq MasterMix (2X) or High-Fidelity PCR Enzyme Mix (Fermentas, ThermoFisher) following the manufacturer’s instructions, with the primers listed in Supplementary Table S2. All other manipulations were performed using standard procedures^43^. DNA was transferred into *P. aeruginosa* cells via electroporation using a Gene Pulser XCell™ electroporator (Bio-Rad) as previously described^30^. The absence of mutations introduced during cloning was verified via DNA sequencing.

An AlgR transcriptional factor overproducer was built by cloning the *algR* gene (PA5261) into the pET28a overexpression system (Novagen) downstream of the T7 RNA polymerase promoter. The *algR* gene was amplified from *P. aeruginosa* PAO1 by PCR using the primers AlgR-up and AlgR-low and High-Fidelity PCR Enzyme Mix. The fragment amplified (747 bp) was cloned into the pGEM-T easy vector and transformed into *E. coli* DH5α. After plasmid isolation using GeneJET Plasmid Miniprep Kit, the plasmid was digested with *Nde*I and *Not*I restriction enzymes and ligated with T4 DNA ligase into the pET28a vector, obtaining plasmid pETS201. Finally, pETS201 was transformed into the Rosetta(DE3) strain for AlgR overproduction and purification.

To produce the AlgRD54N mutant overproducer, the *alg*R gene was specifically mutated by PCR-based site-directed mutagenesis as previously described^19^ using primer pair 1 (see Supplementary Table S3). The mutant gene obtained was cloned into the pGEM-T easy vector, transformed into *E. coli* DH5α cells and verified by DNA sequencing. The *fimS* gene was not included in the cloned fragment to ensure the maximum possible difference in the AlgR phosphorylation state. NdeI and *Not*I restriction enzymes were used for fragment digestion and cloning into the pET28a vector using T4 DNA ligase. Finally, pETS202 was transferred into the Rosetta(DE3) strain for AlgRD54N overproduction and purification.

Complementation vectors for providing extra copies of AlgR and AlgRD54N were constructed by cloning the corresponding genes under the control of their own promoter regions into the pUCP20T vector. First, a band containing the *algR* gene, the neighboring gene *fimS*, and their promoter region was amplified (2286 bp) using primer pair 2 and cloned into pUCP20T, generating pETS203. The *algR* was site-specifically mutagenized as previously described^19^ to produce *algR*D54N using mutagenic primer pair 1 and outer primer pair 2 and cloned into pUCP20T to generate pETS204.

To construct the *algD, algR* and PA1157 transcriptional GFP fusions, 900 bp, 483 bp and 769 bp long fragments encompassing the *algD, algR*, and PA1157 promoter regions were amplified by PCR using primer pairs 3–5; the obtained DNA fragments were cloned into pGEM-T easy and transformed into *E. coli* DH5α cells. *BamHI* and *SmaI* restriction enzymes were used for fragment digestion and for cloning into pETS130-GFP, to generate pETS205, pETS206 and pETS207 plasmids for *algD*, *PA1157* and *algR* promoter regions, respectively.

For AlgR-box mutagenesis in the studied promoters, PCR-based site-directed mutagenesis was used as previously described^19^, using outer primer pairs 6 and 7 for the *nrdAB* and *nrdJab* promoter regions, respectively; mutagenic internal primer pairs 8–10 were used. Two regions of the P*nrdJ* promoter mistakenly identified as AlgR-boxes as artifacts in the bioinformatic search were also mutated using mutagenic primer pairs 11 and 12. For all the positively identified AlgR boxes, the mutant DNA fragments were later cloned into the pGEM-T easy vector and transformed into *E. coli* DH5α cells. BamHI and SmaI restriction enzymes were used for fragment digestion and cloning into pETS130-GFP, to generate pETS208 (for P*nrdA* box 1), pETS209 (for P*nrdJ* box 1) and pETS210 (for P*nrdJ* box 2). For the exact sequence of the mutations introduced, see Fig. 3B.

### Green fluorescent protein-based gene reporter assay

The different *P. aeruginosa* strains were grown in separate Erlenmeyer flasks containing 20 ml LB broth and the specified antibiotic. Flasks were incubated at 37 °C and agitated at 200 rpm. Bacterial growth was monitored by measuring optical density at 550 nm (OD_550_). Upon reaching the desired OD_550_, three independent 1 ml samples were taken from each analyzed strain and centrifuged for 10 minutes at 13000 rpm; the supernatant was removed, and the pellet was washed with PBS 1x containing 2% formaldehyde. Suspensions were left on ice for ten minutes before being centrifuged again, the supernatant removed and PBS 1x added. The fluorescence was then measured after diluting the sample 8 times in PBS 1x, using 96-well plates (Costar® 96-Well Black Polystyrene plate, Corning) on an Infinite 200 Pro Fluorescence Microplate Reader (Tecan).

To determine gene expression during biofilm formation, an aerobic static biofilm was grown on 96-well plates (Nunclon Delta Surface, Thermo Scientific) in LB containing 0.2% glucose. At the desired time, the planktonic cells on the supernatant were removed, and the biofilm was washed three times with PBS 1x and then fixed with 2% formaldehyde. GFP was measured using Infinite 200 Pro Fluorescence Microplate Reader (Tecan). Fluorescence obtained at each time point was compared with fluorescence at 3 h of biofilm formation to calculate the induction factor of the gene expression.

For gene expression measurement during colony formation, 5 μl inocula at an OD_550_ of 0.05 of the corresponding *P. aeruginosa* strains were grown on 6-well plates (Nunclon Delta Surface, Thermo Scientific) containing LB with 1.5% agar and the corresponding antibiotics. Plates were incubated at 37 °C, and GFP expression was measured at different phases of colony formation; fluorescence measurements were performed by using an Infinite 200 Pro Fluorescence Microplate Reader (Tecan).

### Bioinformatic prediction of AlgR binding boxes

To identify putative AlgR binding sites on RNR promoters, a thorough bioinformatics search was conducted. As a first step, we used MEME (MEME suite^44,45^), to generate count matrices characteristic of the AlgR binding box. As the binding sequence is small and somehow flexible^14^, different sources were considered to obtain the AlgR box motif. Three sets of sequences were therefore used: set 1, to obtain a motif characteristic of strong binders, formed by the 50 most enriched sequences in ChIP-seq after AlgR precipitation^12^; set 2, to form a more flexible motif sequence, 25 randomly selected sequences from the enriched group in the same ChIP-seq experiments; and set 3, to capture the variation observed in some experimentally demonstrated boxes, confirmed by a previously published cluster of representative binding sites^14^. Assuming one occurrence of the AlgR box on every sequence, a single 11-bp long motif was obtained from each set (see Supplementary Fig. S1), each one defined by a count matrix.

Using the generated count matrices, we used FIMO (MEME suite^44,46^), to search for AlgR binding sites. We calibrated the search from each count matrix by using a negative control (a 1050 bp-long probe of random DNA with a 67% GC content, to match genomic *P. aeruginosa* DNA) and a positive control (a 1050 bp probe of *algD* promoter spanning all three identified AlgR binding sites^3^, from −900 to +150 bp, counted from the *algD* start codon). For the final search, DNA probes used were 900 bp long for P*nrdJ* and P*nrdD* (from −750 bp to +150 bp, counted from the corresponding start codons) and 1050 bp long for P*nrdA*, given the predicted long 5′ UTR present (from −900 bp to + 150 bp, counted from the *nrdA* start codon).

### AlgR overexpression and purification

AlgR and AlgRD54N proteins were overexpressed in a Rosetta (DE3) *E. coli* strain transformed with pETS201 or pETS202, respectively (Supplementary Table S1). Cells were grown in LB medium with 30 µg ml^−1^ kanamycin and 17 µg ml^−1^ chloramphenicol and incubated at 37 °C with vigorous shaking (250 rpm). When cultures reached an OD_550_ ≈ 0.5, protein overexpression was induced by adding IPTG to a concentration of 1.0 mM (Isopropyl β-D-1-thiogalactopyranoside; Fermentas, Thermo Scientific); cells were cultured at 37 °C for 6 hours and later pelleted by centrifugation.

For preparing the protein extract, the pellet was suspended in 15 ml of AlgR lysis buffer per liter of original culture (50 mM Tris, pH 7.8 at 25 °C; 300 mM NaCl; 20 mM imidazole; 2 mM DTT; 10% glycerol), supplemented with 1 mM PMSF as a protease inhibitor. The resulting suspension was sonicated on ice using a 6 mm conical microtip, until clear, to generate the crude extract (CE). It was centrifuged at 15000 g for 30 minutes at 4 °C, keeping the supernatant as the soluble fraction (SF), which was frozen at −80 °C for long term storage.

AlgR and AlgRD54N were purified from their corresponding SF by Immobilized Metal Affinity Chromatography (IMAC) using a 5 ml His-Trap^TM^ HP column (GE Healthcare) in an FPLC system (BioLogic DuoFlow System, Bio-Rad). First, the column was equilibrated with 5 column volumes (CV) of Buffer A (50 mM Tris-HCl pH 7.8 at 25 °C; 300 mM NaCl; 20 mM imidazole). Protein samples were diluted with buffer A to a concentration of less than 1 mg/ml of total protein content and then injected into the column. A washing step was then carried out with 5 CV of Buffer A, and contaminant proteins were removed with a non-specific elution step using 5 CV of Buffer A with 50 mM imidazole. Finally, the protein was recovered in a specific elution step using 5 CV of Buffer A with 400 mM imidazole. The resulting fractions were analyzed by SDS-PAGE protein electrophoresis and dialyzed against AlgR Binding buffer (20 mM Tris-HCl, pH 7.8 at 25 °C; 120 mM KCl; 2 mM MgCl2; 10% glycerol) and stored at −80 °C (see Supplementary Fig. S5). Protein concentrations were determined by the Bradford assay (Bio-Rad) with crystalline bovine serum albumin as a standard.

### Electrophoretic mobility shift assays (EMSA)

DNA probes for EMSA were produced for analyzing full promoter regions of the *nrdAB* and *nrdJab* operons (P*nrdA* long and P*nrdJ* long bands) or fragments of these promoters (P*nrdA* short 1 and P*nrdA* short 2, as well as P*nrdJ* short 1, P*nrdJ* short 2 and P*nrdJ* short 3; see Fig. 2). DNA probes were also generated for the *nrdDG* operon promoter region (P*nrdD* long band) and the negative control (inner region of non-related *anr* gene) and positive control, using the *algD* operon promoter region (P*algD* band). All probes were generated by amplifying the corresponding region in a first PCR reaction that uses the reverse primer to also add the arbitrary sequence 5′-CTGGGCGTCGTTTTAC-3′ at the 3′ end of every probe (a sequence that we call the M13 complementarity tail) and later applying a second PCR reaction using primer WellRed-M13 to label the probes; WellRed-M13 is a WellRED dye-labeled oligo (Sigma-ALDRICH) coupled to the near-infrared fluorophore D3-phosphoramidite (D3-PA). Resultant probes are hence double-stranded DNA fragments labeled with a single molecule of D3-PA. Primer pairs 13–21 were used for EMSA band generation (see Supplementary Table S3). All wild-type probes were copied from *P. aeruginosa* PAO1 genomic DNA. All probes harboring mutations in putative AlgR-boxes were copied from the corresponding plasmids including mutant promoters (see the DNA manipulation and plasmid construction section).

Purified AlgR or AlgRD54N proteins were used in binding reactions for a total amount of 0, 1, 4 or 10 pmol per reaction. A fixed amount of 100 fmol of DNA was used for all bands. Binding reactions also contained BSA (0.25 µg/reaction) and salmon sperm DNA (1 µg/reaction), as well as 2x-AlgR-binding buffer, added to a final 1x concentration of 20 mM Tris-HCl (pH 7.8 at 25 °C), 100 mM KCl, 2 mM MgCl_2_, 2 mM dithiothreitol, and 10% glycerol. Water was added to every reaction for a final volume of 20 µl. Reactions were incubated at room temperature for 20 minutes before gel electrophoresis.

Electrophoresis was performed in 5% acrylamide gels, prepared with a 37.5:1 proportion of acrylamide:bis-acrylamide and using 5% triethylene glycol as an additive for increased DNA-protein complex stability. Final images were obtained by scanning the gels using the Odyssey Imaging System (LI-COR Biosciences) working in the 700-nm channel.

### Atomic force microscopy (AFM)

DNA probes for AFM studies were generated by PCR from *P. aeruginosa* PAO1 genomic DNA and designed so that binding sites were closer to one of the ends, to easily distinguish binding events. Primer pairs 22–25 were used for generating the DNA probes (see Supplementary Table S3). The length of this probes is higher than 700 bp, to ensure enough DNA for a proper binding to the surface even in the presence of protein. This length is much higher than the expected persistence length of the *P*. aeruginosa DNA, yielding probes that are assumed to bind in stochastic shapes, and will, therefore, be analyzed in large groups to provide statistically significant information. To avoid agarose contamination, when PCR conditions were proved to result in one single amplification band, DNA probes were purified directly from PCR reactions using a GeneJET PCR Purification Kit (Fermentas, ThermoFisher). Purified DNA probes were diluted to 2–4 nM with DNA AFM buffer (10 mM HEPES, pH 7.8 at 25 °C; 5 mM MgCl_2_; 50 mM NaCl). Ten microliters of DNA solution were pipetted onto a freshly cleaved mica and allowed to deposit for 1 min. The mica surface was then rinsed with 200 µl of MilliQ water and dried under a nitrogen stream. For the DNA-protein complex images, protein (AlgR/LexA) was previously mixed with the DNA fragments to a molar ratio of 3:1; the complex was incubated for 20 minutes at room temperature, and 10 µl of the solution was deposited on freshly cleaved mica and allowed to deposit for 4 minutes before rinsing and drying. Topographic images were obtained with a commercial AFM system (CypherTM, Asylum Research) in conventional dynamic mode. A PPP-CONTR (Nanosensors) tip was used, with a nominal spring constant of ~ 0.3 N/m and tip radius of ~7 nm, scanning in ambient conditions using small oscillation amplitudes (~20 nm). Image resolution was not lower than 6 nm/pixel since this is close to the tip radius curvature. AFM image processing and determination of DNA length were carried out using WSxM 5.0 develop 7.0 (WSxM solutions).

### Availability of Data and Materials

All data generated or analyzed during this study are included in this manuscript and it supplementary information files, or is available upon request.

## Electronic supplementary material

Supplementary Information
