# Supplementary material for: Regulation of ribonucleotide synthesis by the Pseudomonas aeruginosa two-component system AlgR in response to oxidative stress
Source: Sci Rep. 2017 Dec 20;7:17892. doi: 10.1038/s41598-017-17917-7 (PMC5738425; doi:10.1038/s41598-017-17917-7)
Supplement: Supplementary file 1 — Supplementary Information [file 41598_2017_17917_MOESM1_ESM.pdf]

Regulation of ribonucleotide synthesis by the *Pseudomonas aeruginosa* two-component system AlgR in response to oxidative stress

Anna Crespo[1¶], Lucas Pedraz[1¶], Marc Van Der Hofstadt[2], Gabriel Gomila[2], Eduard Torrents[1*]

[1] Bacterial Infections: Antimicrobial Therapies, Institute for Bioengineering of Catalonia (IBEC), The Barcelona Institute of Science and Technology, Barcelona, Spain.

[2] Nanoscale Bioelectrical Characterization, Institute for Bioengineering of Catalonia (IBEC), The Barcelona Institute of Science and Technology, Barcelona, Spain.

*: Corresponding author; e-mail: etorrents@ibecbarcelona.eu (ET)

¶: AC and LP, listed in alphabetical order, contributed equally to this work.

Supplementary Fig. S1. Count matrices for AlgR-box identification. Count matrices were generated by FIMO search using three different sets of sequences containing AlgR binding spots (see Materials and Methods). Matrices are adjusted for a box size of 11 bp, represented in rows, and the bases are expressed in columns in the order A–C–G–T; each matrix is accompanied by its corresponding HMM logo.

Set 1: 50 best hits in ChIP-seq

30	0	19	1
0	50	0	0
1	49	0	0
6	0	42	2
2	2	0	46
0	5	0	45
0	46	3	1
5	0	44	1
0	0	0	50
0	50	0	0
6	6	32	6

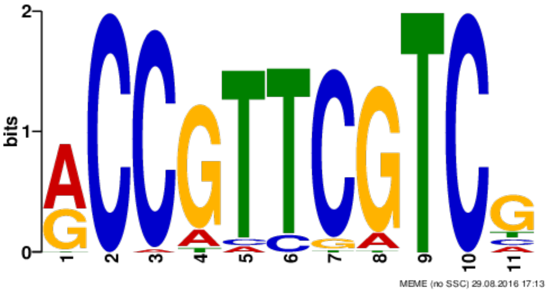

Set 2: 25 random hits in ChIP-seq

4	2	6	11
14	3	4	2
0	23	0	0
0	23	0	0
1	0	21	1
0	3	0	20
0	6	0	17
0	19	4	0
5	0	18	0
1	0	0	22
0	23	0	0

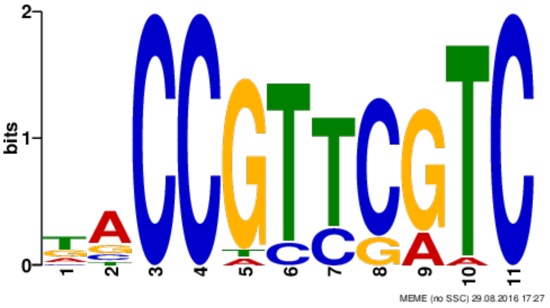

Set 3: Published sequences

3	4	3	1
0	11	0	0
0	11	0	0
0	1	10	0
0	0	0	11
0	2	1	8
0	6	3	2
1	0	8	2
0	0	2	9
0	11	0	0
1	6	3	1

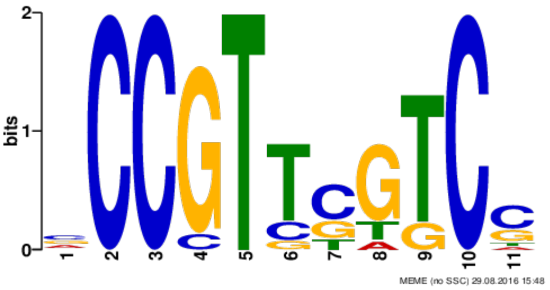

Supplementary Fig. S2. Original EMSA images used to generate Figure 3A.

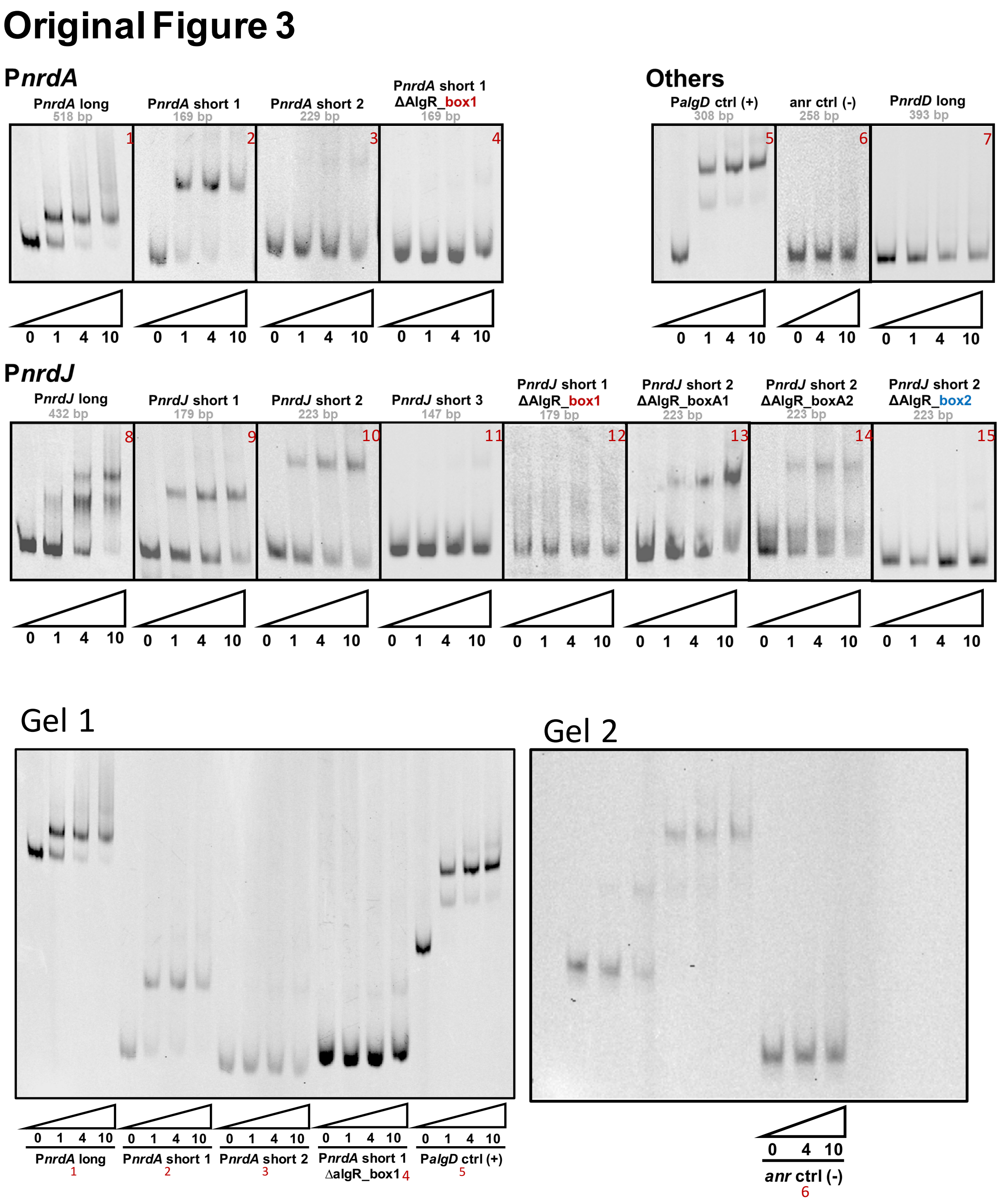

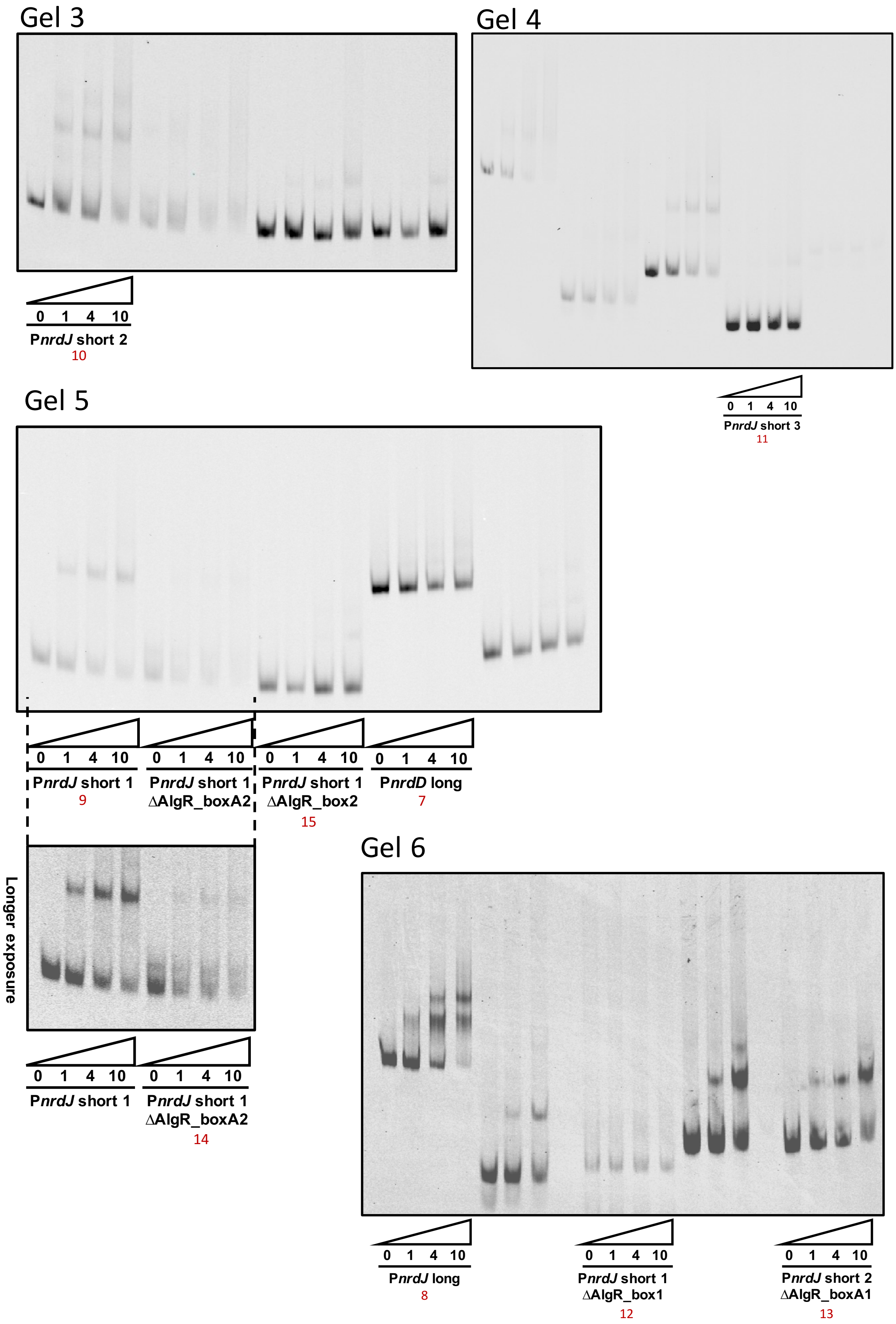
Gel 3
0 1 4 10
PnrdJ short 2
10
Gel 4
0 1 4 10
PnrdJ short 3
11
Gel 5
0 1 4 10
PnrdJ short 1
9
0 1 4 10
PnrdJ short 1 ΔAlgR_boxA2
0 1 4 10
PnrdJ short 1 ΔAlgR_box2
15
0 1 4 10
PnrdD long
7
Longer exposure
0 1 4 10
PnrdJ short 1
0 1 4 10
PnrdJ short 1 ΔAlgR_boxA2
14
Gel 6
0 1 4 10
PnrdJ long
8
0 1 4 10
PnrdJ short 1 ΔAlgR_box1
12
0 1 4 10
PnrdJ short 2 ΔAlgR_boxA1
13

Supplementary Fig. S3. AlgR – DNA binding affinities. EMSA assays for P*nrdA*, P*nrdJ,* and *PalgD* promoter long bands. (**A**), A wide array of concentrations was used to illustrate different binding affinities (shown below the figures; numbers represent protein amount in pmol). Different boxes involved in bindings are shown in the table below, (**B**). Most conserved base pairs are underlined, and cytosine in position 7, which is described to distinguish weak and strong binding sites, is marked in gray.

A

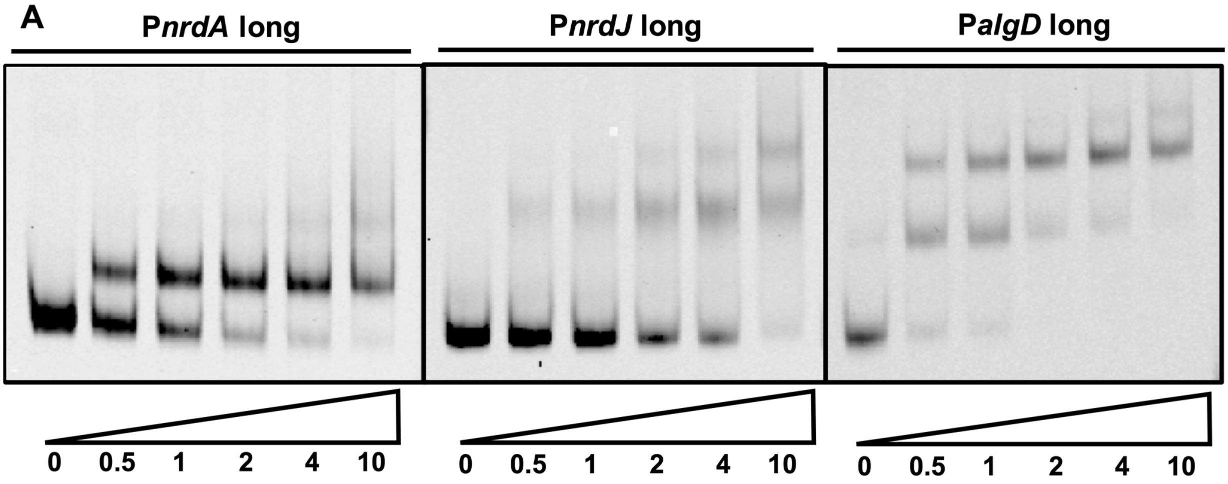

B

Binding site	Sequence	Orientation	Strength	Source
P*nrdA* box 1	GCCATTCGTCG	3'-5' ←	Strong	This work
P*nrdJ* box 1	GCCGCCGGTCC	5'-3' →	Weak	This work
P*nrdJ* box 2	GCCGGCTGTCT	5'-3' →	Weak	This work
P*algD* RB1	ACCGTTCGTCC	5'-3' →	Strong	1, 2
P*algD* RB2	ACCGTTCGTCT	5'-3' →	Strong	1, 2
P*algD* RB3	GCCGTTTGTCC	3'-5' ←	Weak	1, 2
P*algC* ABS1	CCCGTTCGTCG	5'-3' →	Strong	3
P*algC* ABS2	TCCGTTGTTCC	5'-3' →	Weak	3
P*algC* ABS3	ACCGTGCGTCG	5'-3' →	Strong	3

(1) Kato, J. and A. M. Chakrabarty (1991). "Purification of the regulatory protein AlgR1 and its binding in the far upstream region of the algD promoter in Pseudomonas aeruginosa." Proc Natl Acad Sci U S A 88(5): 1760-1764.

(2) Mohr, C. D., et al. (1991). "AlgR, a response regulator controlling mucoidy in Pseudomonas aeruginosa, binds to the FUS sites of the algD promoter located unusually far upstream from the mRNA start site." J Bacteriol 173(16): 5136-5143.

(3) Zielinski, N. A., et al. (1991). "Characterization and regulation of the Pseudomonas aeruginosa algC gene encoding phosphomannomutase." J Biol Chem 266(15): 9754-9763.

Supplementary Fig. S4. Cooperative regulation of AlgZR and Anr/Dnr systems on RNR class II. Gene reporter assay for the P*nrdJ* promoter fused to GFP, during anaerobic liquid cultures, grown to OD_{550} = 2.0. The cooperative action of these two systems is explored by combining a Δ*algR* background with the mutation of the Anr/Dnr box on P*nrdJ*. Values are averages from at least three independent experiments; error bars show positive standard deviation. Asterisks (*) indicate statistically significant differences from the wild-type strain harboring P*nrdJ* wild-type promoter (*p*-value less than 0.05 in pairwise T-tests). Shortened names are used (see Table S1).

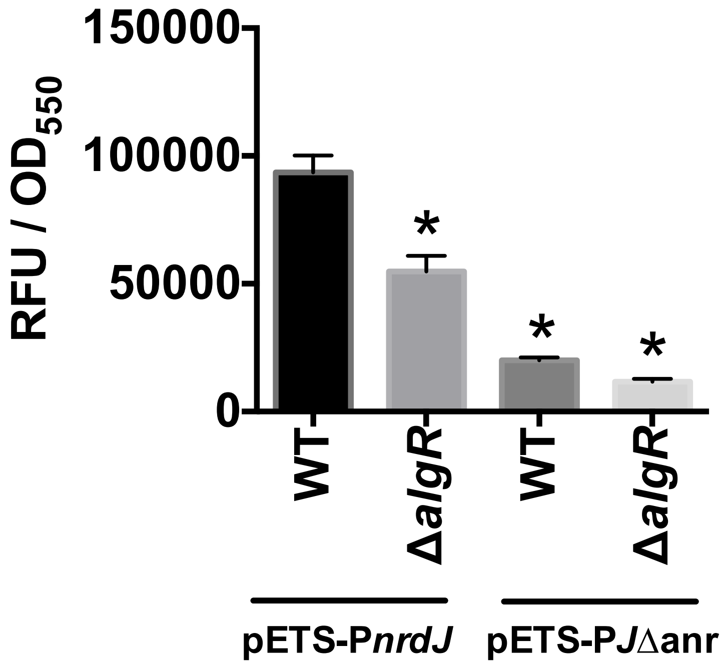

Supplementary Fig. S5. Protein overexpression and purification. Coomassie blue-stained gel showing SDS-PAGE analysis of AlgR wild type and AlgRD54N overexpression. MW, molecular weight marker; CE, crude extract; FT, flow through; UN, nonspecific elution step; P, protein recovered after specific elution step. Molecular weights of the standards are indicated.

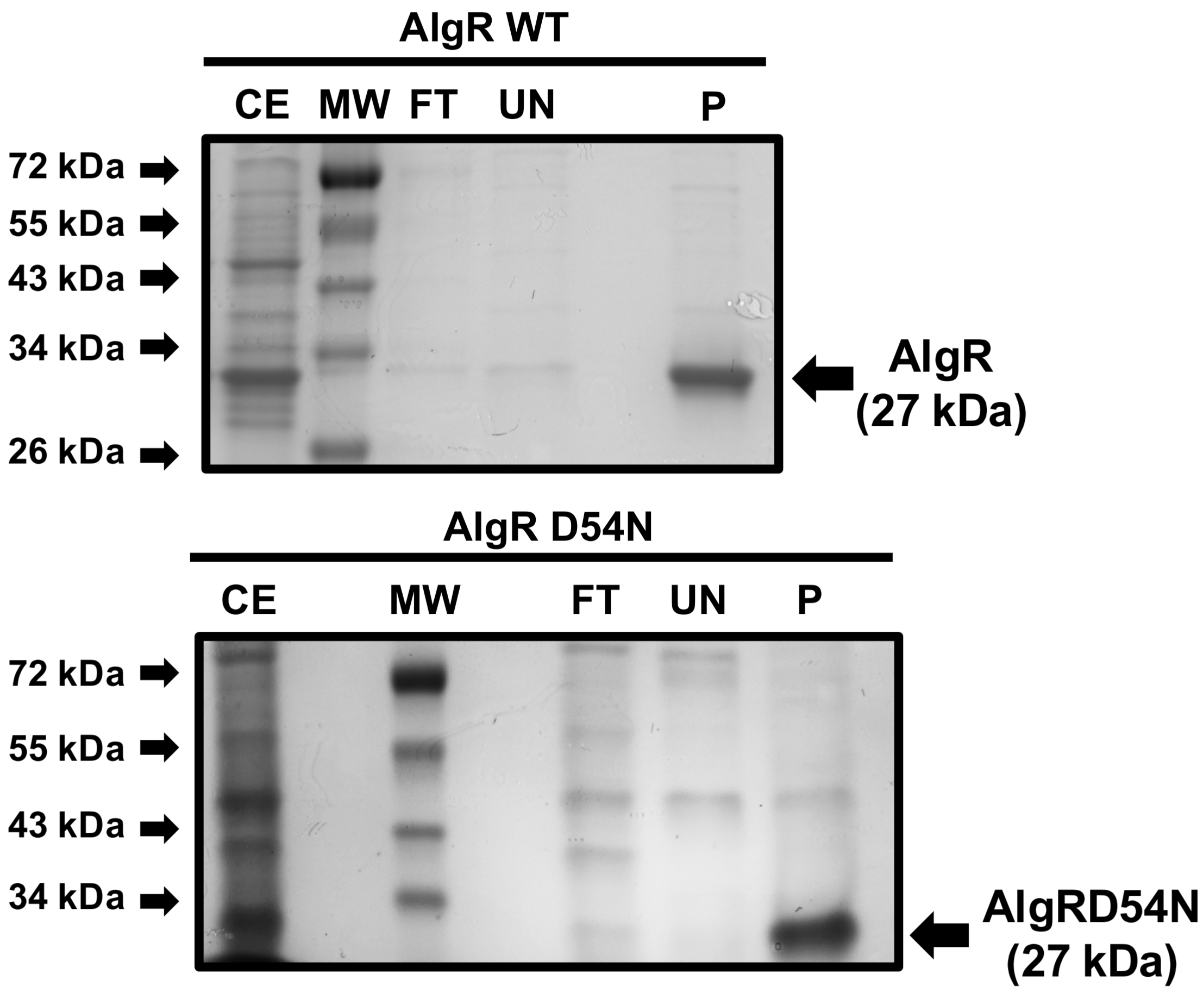

Supplementary Table S1. Bacterial strains and plasmids used in this study. For each element, a general description is provided, together with an alternative self-explanatory name which will be commonly used in figures to make interpretation of the data easier for the reader. Throughout all the paper, a P before the name of a gene indicates the promoter controlling this gene (e.g., P*nrdA* for *nrdAB* operon promoter).

Name	Referred as…	Description	Source
**Plasmids**			
pGEM-T easy	pGEM-T easy	A/T cloning vector; Amp^R	Promega
pUCP20T	pUCP20T	Broad-host-range vector; Amp^R	(1)
pET28a	pETS28a	Vector for His_6-tagged protein overexpression; Kn^R	Laboratory
pETS130-GFP	pETS130	Broad host range, promoterless GFP; Gm^R	(2)
pETS134	pETS-P*nrdA*	pETS130 derivative carrying *nrdA* promoter; Gm^R	(2)
pETS136	pETS-P*nrdD*	pETS130 derivative carrying *nrdD* promoter; Gm^R	(2)
pETS180	pETS-P*nrdJ*	pETS130 derivative carrying *nrdJ* promoter; Gm^R	(3)
pETS191	pETS-P*J*Δdnr	pETS130 derivative carrying Anr/Dnr box mutating in P*nrdJ*; Gm^R	(4)
pETS201	pETS201	pET28a derivative carrying *algR*, AlgR overproducer, Kn^R	This work
pETS202	pETS202	pET28a derivative carrying algRD54N, AlgRD54N overproducer, Kn^R	This work
pETS203	pUCP-AlgR	pUCP20T derivative carrying the *algR* gene; Cb^R	This work
pETS204	pUCP-D54N	pUCP20T derivative carrying the *algRD54N* gene; Cb^R	This work
pETS205	pETS-P*algD*	pETS130 derivative carrying *algD* promoter; Gm^R	This work
pETS206	pETS-P1157	pETS130 derivative carrying *PA1157* promoter; Gm^R	This work
pETS207	pETS-P*algR*	pETS130 derivative carrying *algR* promoter; Gm^R	This work
pETS208	pETS-P*A*Δbox1	pETS130 derivative carrying AlgR-box1 mutation in P*nrdA,* Gm^R	This work
pETS209	pETS-P*J*Δbox1	pETS130 derivative carrying AlgR-box1 mutation in P*nrdJ,* Gm^R	This work
pETS210	pETS-P*J*Δbox2	pETS130 derivative carrying AlgR-box2 mutation in P*nrdJ,* Gm^R	This work
pETS211	pETS-P*J*Δbox1+2	pETS130 derivative carrying AlgR-box1 and AlgR-box2 mutation in P*nrdJ,* Gm^R	This work
**Strains**			

E. coli			
DH5α	DH5α	*recA1 endA1 hsdR17 supE44 thi-1 relA1 Δ(lacZYA-argF)U169 deoR Φ80dlacZM15*	Laboratory
Rossetta(DE3)	Rosetta	F^{-} *ompT* $hsdS_B$ (r_B^{-} m_B^{-}) *gal dcm (DE3) pRARE (CamR)*	Merck
P. aeruginosa			
PAO1	PAO1 WT	Wild-type (ATCC 15692 / CECT 4122) - Spanish Type Culture Collection	Lab strain
PW9855	PAO1 Δ*algR*	*P. aeruginosa* PAO1 *algR*::*IS*phoA/hah; Tc^R	(5)
PAOMA	PAO1 Δ*mucA*	*P. aeruginosa mucA* strain	A. Oliver Lab

1. **West SE, Schweizer HP, Dall C, Sample AK, Runyen-Janecky LJ.** 1994. Construction of improved *Escherichia-Pseudomonas* shuttle vectors derived from pUC18/19 and sequence of the region required for their replication in *Pseudomonas aeruginosa*. Gene **148:**81-86.
2. **Sjöberg BM, Torrents E.** 2011. Shift in ribonucleotide reductase gene expression in *Pseudomonas aeruginosa* during infection. Infect Immun **79:**2663-2669.
3. **Crespo A, Pedraz L, Torrents E.** 2015. Function of the *Pseudomonas aeruginosa* NrdR Transcription Factor: Global Transcriptomic Analysis and Its Role on Ribonucleotide Reductase Gene Expression. PLoS One **10:**e0123571.
4. **Crespo A, Pedraz L, Astola J, Torrents E.** 2016. *Pseudomonas aeruginosa* Exhibits Deficient Biofilm Formation in the Absence of Class II and III Ribonucleotide Reductases Due to Hindered Anaerobic Growth. Front Microbiol **7:**688.
5. **Jacobs MA, Alwood A, Thaipisuttikul I, Spencer D, Haugen E, Ernst S, Will O, Kaul R, Raymond C, Levy R, Chun-Rong L, Guenthner D, Bovee D, Olson MV, Manoil C.** 2003. Comprehensive transposon mutant library of *Pseudomonas aeruginosa*. Proc Natl Acad Sci U S A **100:**14339-14344.

Supplementary Table S2. **Primers and probes used in this study.**

Name	Sequence (5'→3')	Application
M13-dir	GTTTTCCCAGTCACGAC	Check-Cloning
M13-rev	CAGGAAACAGCTATGACC	Check-Cloning
pUCP20T-up	CCTCTTCGCTATTACGCCAG	Cloning
pUCP20T-low	TCCGGCTCGTATGTTGTGTG	Cloning
pBBR1-up	CATCGCAGTCGGCCTATTGG	Cloning
pBBR1-low	CACTTTATGCTTCCGGCTCG	Cloning
AlgR-up	ACATATGAATGTCCTGATTGTCGATG	AlgR overproducer
AlgR-low	ATCAGAGCTGATGCATCAGAC	AlgR overproducer
AlgRD54N-up	GCGGATGTTCAGCAGGAC	AlgRD54N overproducer
AlgRD54N-low	GTCCTGCTGAACATCCGC	AlgRD54N overproducer
PfimSalgR-up	GGATCCTGTCTTCCTGGTTGTCCTTGTT	P*fimSalgR* cloning / AlgR complementation
PalgR-SmaI-GFP-low	TTCCCGGGCTTGAATCGGAT	P*fimSalgR* cloning
PA1157-up	AAGGATCCGGTATGCATGGGTGGGTATC	*PA1157* promoter cloning
PA1157-low	AACCCGGGTTCTTGCTCCACACAGCCTC	*PA1157* promoter cloning
PnrdA-BamHI-EcoRI-GFP-up	AGGATCCGAATTCTTGCTCCACACAGCCTC	P*nrdAB* cloning / EMSA P*nrdA* long / AFM
PnrdA-SmaI-GFP-low	ACCCGGGTTCTCGCGTGTGGTGTCG	P*nrdAB* cloning / AFM
PnrdA-EXT-low-M13	CTGGGCGTCGTTTTACGGCTCCTTGCGATGAG	EMSA P*nrdA* long
PnrdA-AlgR-EMSA-up	TACATATTGTGGGTAGGGTG	EMSA P*nrdA* short 1
PnrdA-AlgR-EMSA-low-M13	CTGGGCGTCGTTTTACGGATAAAGTGTGGGTCTTCT	EMSA P*nrdA* short 1
PnrdA-EMSA-up	TTTCCCCCAGACTGTCAC	EMSA P*nrdA* short 2
PnrdA-EMSA-low-M13	CTGGGCGTCGTTTTACTCAGAGTGGTCCGTGCG	EMSA P*nrdA* short 2
PnrdJ-AlgR-BamHI-EMSA-up	GGATCCTACGGGTTGCGCCATA	P*nrdJ* promoter cloning
PnrdJ-SmaI-GFP-low	AACCCGGGGACTGCGTTGCGTCTGTC	P*nrdJ* promoter cloning / AFM
PnrdJ-BamHI-GFP-up	GGATCCCGCGCCCAGCTGAAGGCC	EMSA P*nrdJ* long
PnrdJ-EXT-low-M13	CTGGGCGTCGTTTTACGGCCACCGTACGCAAC	EMSA P*nrdJ* long
PnrdJ-AlgR-EMSA-up	TACGGGTTGCGCCATA	EMSA P*nrdJ* short 1
PnrdJ-AlgR-EMSA-low-M13	CTGGGCGTCGTTTTACTTCGCTGAGGGTGTCG	EMSA P*nrdJ* short 1

PnrdJ-mid-up	CCGACACCCTCAGCGAAG	EMSA P*nrdJ* short 2
PnrdJ-mid-low-M13	CTGGGCGTCGTTTTACAGACAACCTTAGTCATCGG	EMSA P*nrdJ* short 2
PnrdJ-EMSA-up	TCCCGATGACTAAGGTTGTC	EMSA P*nrdJ* short 3
PnrdJ-EMSA-low-M13	CTGGGCGTCGTTTTACCTGATTAACCTCCCGATGG	EMSA P*nrdJ* short 3
PnrdJ-AFM-up	GCGCAAGTTCGTCAATTTCG	AFM
PnrdD-BamHI-GFP-up	AGGATCCCGCGACGCCCATTTC	EMSA P*nrdD* long
PnrdD-EMSA-low-M13	CTGGGCGTCGTTTTACCTTGAGCAGGGTGGCC	EMSA P*nrdD* long
PalgD-BamHI-GFP-up	GGATCCCTCCTCTTTCGGCAC	P*algD* cloning / EMSA positive control
PalgD-low-M13	CTGGGCGTCGTTTTACTTCCTTAATCTTCGACCCA	EMSA positive control / AFM
PalgD-SmaI-GFP-low	CCCGGGAGATGCTGATTCGCATC	P*algD* cloning
PalgD-BamHI-AFM-up	TGGATCCCCCTATCGACTGGAAATGG	AFM
Anr-EcoRI-up	GAATTCATGGCCGAAACCATCAAG	EMSA negative control
Anr-low-M13	CTGGGCGTCGTTTTACGCATCGGTGATGCTGAAG	EMSA negative control
DinB-AFM-up	CTGGTGATGCTGGTCGTG	AFM
DinB-low-M13	CTGGGCGTCGTTTTACCAGCTCCCGCAACCAC	AFM
PnrdA-mutAlgR1-up	GCTTCGCCTAACATTCTCCAGCGCTG	Mutagenesis P*nrdA* box1
PnrdA-mutAlgR1-low	TGTTAGGCGAAGCCCTCGGAAAGC	Mutagenesis P*nrdA* box1
PnrdJ-mutAlgR1-up	GGTTGCCGTAACGGTCTGCA	Mutagenesis P*nrdJ* box1
PnrdJ-mutAlgR1-low	CAGACCGTTACGGCAACCT	Mutagenesis P*nrdJ* box1
PnrdJ-mutAlgR2-up	GCTCTGAAAACTAGTTCCTGATATCCGC	Mutagenesis P*nrdJ* boxA1
PnrdJ-mutAlgR2-low	GCGCGGATATCAGGAACTAGTTT	Mutagenesis P*nrdJ* boxA1
PnrdJ-mutAlgR3-up	ATGGCCGCGAACGCTTGAGCG	Mutagenesis P*nrdJ* boxA2
PnrdJ-mutAlgR3-low	CGCTCAAGCGTTCGCGGCCAT	Mutagenesis P*nrdJ* boxA2
PnrdJ-mutAlgR4-up	CGAATTTGAAGGCTTAATGGAAAAGC	Mutagenesis P*nrdJ* box2
PnrdJ-mutAlgR4-low	TTCCATTAAGCCTTCAAATTCGC	Mutagenesis P*nrdJ* box2
WellRed-M13	[D3-PA]GTCACTGGGCGTCGTTTTAC	EMSA band infrared labelling

Supplementary Table S3. PCR reactions and primer pairs used.

Primer pair	Forward primer	Reverse primer	Application
1	AlgR-D54N-up	AlgR-D54N-low	AlgR D54N directed mutagenesis
2	PfimSalgR-up	AlgR-low	AlgR complementation plasmids
3	PalgD-BamHI-GFP-up	PalgD-SmaI-GFP-low	P*algD*::*gfp* transcriptional fusion
4	PfimSalgR-up	PalgR-SmaI-GFP-low	P*algR*::*gfp* transcriptional fusion
5	PA1157-up	PA1157-low	P_{PA1157}::gfp transcriptional fusion
6	PnrdA-BamHI-EcoRI-GFP-up	PnrdA-SmaI-GFP-low	Outer primers in P*nrdA* promoter
7	PnrdJ-AlgR-BamHI-GFP-up	PnrdJ-SmaI-GFP-low	Outer primers in P*nrdJ* promoter
8	PnrdA-mutAlgR1-up	PnrdA-mutAlgR1-low	P*nrdA* AlgR box 1 mutagenesis
9	PnrdJ-mutAlgR1-up	PnrdJ-mutAlgR1-low	P*nrdJ* AlgR box 1 mutagenesis
10	PnrdJ-mutAlgR4-up	PnrdJ-mutAlgR4-low	P*nrdJ* AlgR box 2 mutagenesis
11	PnrdJ-mutAlgR2-up	PnrdJ-mutAlgR2-low	P*nrdJ* AlgR box A1 mutagenesis
12	PnrdJ-mutAlgR3-up	PnrdJ-mutAlgR3-low	P*nrdJ* AlgR box A2 mutagenesis
13	PnrdA-BamHI-EcoRI-GFP-up	PnrdA-EXT-low-M13	EMSA P*nrdA* long band
14	PnrdA-AlgR-EMSA-up	PnrdA-AlgR-EMSA-low-M13	EMSA P*nrdA* short 1 band
15	PnrdA-EMSA-up	PnrdA-EMSA-low-M13	EMSA P*nrdA* short 2 band
16	PnrdJ-BamHI-GFP-up	PnrdJ-EXT-low-M13	EMSA P*nrdJ* long band
17	PnrdJ-AlgR-EMSA-up	PnrdJ-AlgR-EMSA-low-M13	EMSA P*nrdJ* short 1 band
18	PnrdJ-mid-up	PnrdJ-mid-low-M13	EMSA P*nrdJ* short 2 band
19	PnrdJ-EMSA-up	PnrdJ-EMSA-low-M13	EMSA P*nrdJ* short 3 band
20	PalgD-BamHI-GFP-up	PalgD-low-M13	EMSA P*algD* positive control band
21	Anr-EcoRI-up	Anr-low-M13	EMSA *anr* negative control band
22	PnrdA-BamHI-EcoRI-GFP-up	PnrdA-SmaI-GFP-low	AFM P*nrdA* probe
23	PnrdJ-AFM-up	PnrdJ-SmaI-GFP-low	AFM P*nrdJ* probe
24	PalgD-BamHI-AFM-up	PalgD-low-M13	AFM P*algD* probe
25	PdinB-AFM-up	PdinB-low-M13	AFM P*dinB* probe
